# *Salmonella* effector SopD promotes plasma membrane scission by inhibiting Rab10

**DOI:** 10.1038/s41467-021-24983-z

**Published:** 2021-08-04

**Authors:** Kirsten C. Boddy, Hongxian Zhu, Vanessa M. D’Costa, Caishuang Xu, Ksenia Beyrakhova, Miroslaw Cygler, Sergio Grinstein, Etienne Coyaud, Estelle M. N. Laurent, Jonathan St-Germain, Brian Raught, John H. Brumell

**Affiliations:** 1 Cell Biology Program, Hospital for Sick Children, Toronto, ON Canada; 2 Institute of Medical Science, University of Toronto, Toronto, ON Canada; 3 Department of Molecular Genetics, University of Toronto, Toronto, ON Canada; 4 Department of Biochemistry, Microbiology and Immunology, University of Ottawa, Ottawa, ON Canada; 5 Centre for Infection, Immunity and Inflammation, University of Ottawa, Ottawa, Canada; 6 Department of Biochemistry, Microbiology and Immunology, University of Saskatchewan, Saskatoon, SK Canada; 7 Department of Biochemistry, University of Toronto, Toronto, ON Canada; 8 Princess Margaret Cancer Centre, University Health Network, Toronto, ON Canada; 9 Department of Medical Biophysics, University of Toronto, Toronto, ON Canada; 10 SickKids IBD Centre, Hospital for Sick Children, Toronto, ON Canada

**Keywords:** Membrane fission, Phagocytosis, Bacterial pathogenesis, Cellular microbiology

## Abstract

*Salmonella* utilizes translocated virulence proteins (termed effectors) to promote host cell invasion. The effector SopD contributes to invasion by promoting scission of the plasma membrane, generating *Salmonella*-containing vacuoles. SopD is expressed in all *Salmonella* lineages and plays important roles in animal models of infection, but its host cell targets are unknown. Here we show that SopD can bind to and inhibit the small GTPase Rab10, through a C-terminal GTPase activating protein (GAP) domain. During infection, Rab10 and its effectors MICAL-L1 and EHBP1 are recruited to invasion sites. By inhibiting Rab10, SopD promotes removal of Rab10 and recruitment of Dynamin-2 to drive scission of the plasma membrane. Together, our study uncovers an important role for Rab10 in regulating plasma membrane scission and identifies the mechanism used by a bacterial pathogen to manipulate this function during infection.

## Abstract (web summary)

*Salmonella* secretes the effector protein SopD into the host cell cytoplasm, leading to scission of the plasma membrane through unclear mechanisms. Here, Boddy et al. show that SopD binds to and inhibits the small GTPase Rab10, thus promoting removal of Rab10 and recruitment of dynamin-2 to drive plasma membrane scission.

## Introduction

*Salmonella enterica* serovar Typhimurium (*S*. Typhimurium) is an intracellular bacterial pathogen and a major cause of foodborne gastroenteritis in humans^1^. *S*. Typhimurium is capable of invading and replicating in a wide variety of cell types, by using type 3 secretion systems (T3SSs) to translocate virulence factors called effectors into host cells^2^. The *S*. Typhimurium genome encodes two distinct T3SS’s within *Salmonella* pathogenicity islands (SPI)-1 and SPI-2. The SPI-1 T3SS secretes effectors that induce cytoskeletal rearrangements and changes in phosphoinositide dynamics, which drive the internalization of the bacteria into a compartment known as the Salmonella-containing vacuole (SCV)^3^. Subsequently, the SPI-2 encoded T3SS translocates effectors across the SCV membrane to promote intracellular growth and cell-to-cell spread^4–6^.

*Salmonella* T3SS effectors can act cooperatively to initiate specific stages of infection^7,8^. For example, the effectors SopB and SopD work together to drive bacterial internalization, by promoting plasma membrane scission and the generation of SCVs at invasion sites^9^. SopB is a lipid phosphatase that contributes to plasma membrane scission and the generation of SCVs by eliminating its primary substrate in host cells, phosphatidylinositol 4,5-bisphosphate (PI(4,5)P_2_)^10–14^. SopD is conserved in all *Salmonella* lineages and contributes to gastroenteritis^15–17^ and systemic disease models of animal infection^18^; however, its host cell target(s) and mechanism of action have not been defined^9^.

SopD shares 42% sequence identity with another effector, SopD2^19^, which is secreted by the SPI-2 T3SS and is involved in later stages of infection. SopD2 binds to and inactivates several Rab GTPases through multiple mechanisms^20–22^. Whether SopD can also modulate Rab GTPase function is unknown. Here, we demonstrate that SopD binds to and inhibits the activity of Rab10. Furthermore, we show that Rab10 inhibition by SopD contributes to plasma membrane scission and bacterial internalization into SCVs during infection of host cells.

## Results

### SopD is a bacterial GAP for Rab10

**Fig. 1:**
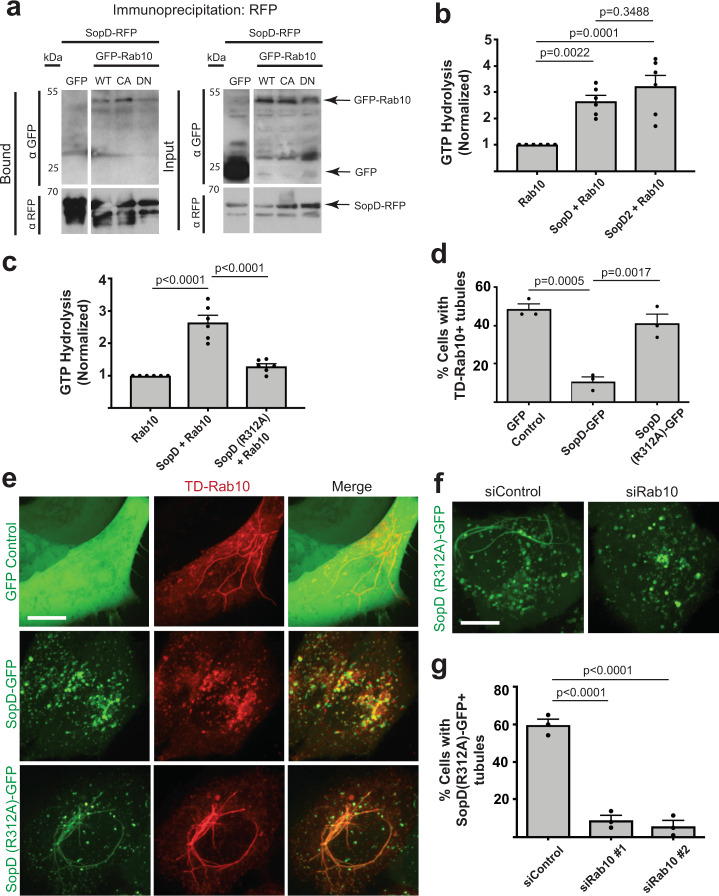
SopD is a bacterial GAP for Rab10. **a** Co-immunoprecipations. HEK923T cells were co-transfected with SopD-RFP and GFP-Rab10 variants, and lysates were precipitated with anti-RFP-Trap affinity resin. Western blot analysis was performed on elutions. **b** Purified Rab10 (8 µM) was incubated alone and with SopD or SopD2 (8 µM). GTP hydrolysis as measured by a malachite green assay, is represented relative to Rab10 alone. GTP hydrolysis was measured as indicated in the Methods section. Values represent relative GTP hydrolysis and are the mean ± S.E.M. of six technical replicates from three independent determinations with multiple protein preparations. Statistical analysis was performed with a one-way ANOVA. **c** Purified Rab10 was incubated alone, with SopD or the SopD catalytic mutant (R312A) (8 µM) and the GTP hydrolysis is represented relative to Rab10 alone. GTP hydrolysis was measured as indicated in the Methods section. Values represent relative GTP hydrolysis and are the mean ± S.E.M. of 6 technical replicates from three independent determinations with multiple protein preparations. Statistical analysis was performed with a one-way ANOVA. **d** Quantification of the number of Henle 407 cells co-transfected with TD-Rab10 and GFP control, SopD, or SopD(R312A)-GFP tagged constructs that have TD-Rab10^+^ tubules. In three independent experiments at least 50 cells were scored for the presence or absence of TD-Rab10^+^ tubules. Data are means ± S.E.M. Statistical analysis was performed with a one-way ANOVA. **e** Representative images of Henle 407 cells co-transfected with TD-Rab10 and GFP control, SopD, or SopD(R312A)-GFP tagged constructs from (**d**). Scale Bar, 15 μm. **f** Representative images of Henle 407 cells that were transfected with Control siRNA or Rab10 siRNA #1 (see methods) and then SopD(R312A)-GFP 24 h later. Scale Bar, 15 μm. **g** Henle 407 cells were transfected with either Control siRNA, Rab10 siRNA #1 or Rab10 siRNA #2 (see “methods”) and then SopD(R312A)-GFP 24 h later. Data represent quantification of the number of transfected cells with SopD(R312A)^+^ tubules. In three independent experiments at least 100 cells were scored. Data are means ± S.E.M. Statistical analysis was performed with a one-way ANOVA. Images are representative of at least three independent experiments. SCV *Salmonella*-containing vacuole, WT wild type, CA constitutively active, DN dominant negative, RFP red fluorescent protein, GFP green fluorescent protein, TD TdTomato. Source data are included in source data file.

Immunoprecipitation coupled with mass spectrometry (IP-MS)^23,24^ was used to identify SopD host cell binding partners in HEK 293 Flp-In T-REx cells. Using this approach, 120 high confidence interactors were identified (Supplementary Data 1: endocytic trafficking related hits are shown in Supplementary Fig. 1). Amongst the high confidence interactors was the small GTPase Rab10. To validate the SopD-Rab10 interaction, HEK 293T cells were co-transfected with SopD-RFP and wild type (WT), dominant negative (T23N) or constitutively active (Q68L) GFP-Rab10 mutants^25,26^. SopD co-immunoprecipitated WT Rab10 and both Rab10 mutants (Fig. 1a).

Proteins that bind the active form of GTPases often function as either effectors or their GTPase activating proteins (GAPs), which terminate their activity by promoting GTP hydrolysis. SopD2 was previously shown to inactivate Rab8, Rab10 and Rab32 in vitro through a direct GAP activity^21^. SopD2 is thought to mimic eukaryotic GAPs, utilizing a catalytic arginine residue (R315) located near its C-terminus^21^. Mutation of this residue to alanine (R315A) causes a loss in SopD2 GAP activity towards Rab8, 10, and 32 as determined by in vitro biochemical assays^21^.

We identified a putative catalytic arginine residue (R312) in the C-terminus of SopD, (Supplementary Fig. 2) suggesting it may also have GAP activity. An in vitro GAP assay performed with purified SopD and Rab10 revealed that SopD accelerates the intrinsic GTP hydrolysis of Rab10, similar to SopD2 (Fig. 1b). The GAP activity of SopD requires R312, since a SopD mutant (R312A) lacking this residue was incapable of accelerating Rab10 GTP hydrolysis (Fig. 1c). Therefore, SopD can function as a GAP for Rab10 in vitro.

### SopD is sufficient to disrupt Rab10^+^ tubules in the absence of infection

Rab10 localizes to tubules in multiple eukaryotic cell types and mediates their formation via its effectors^27,28^. Therefore, we tested the impact of SopD expression on Rab10 localization in control (uninfected) cells. Since SopD loses association with membranes during fixation^9^, we tested localization in live epithelial cells. TdTomato (TD)-Rab10 localized to endosomes and long tubules in ~50% of cells, when co-expressed with a GFP control plasmid (Fig. 1d, e). Co-expression of SopD-GFP and TD-Rab10 led to colocalization on punctate structures accompanied by the loss of TD-Rab10 localization on tubules. In contrast, the SopD GAP mutant (R312A) colocalized with TD-Rab10 but did not disrupt Rab10 localization to tubules. Thus, SopD can interfere with Rab10 localization in epithelial cells, independently of other *S*. Typhimurium effectors and in a manner that requires its GAP activity.

SopD2 was previously shown to have GAP activity towards Rab10 in vitro^21^. However, the expression of either SopD2 or its GAP mutant (R315A) did not impact Rab10 localization to tubules (Supplementary Fig. 3). Thus, SopD can specifically alter Rab10 tubule localization in transfected cells.

SopD was previously shown to bind to host cell membranes during infection but the mechanisms for this binding are not known^9^. To test a role for Rab10 in binding of SopD to host cell membranes, siRNA-mediated knockdown of Rab10 (confirmed by Western blot or qRT-PCR, Supplementary Fig. 4a–c) was performed with two separate siRNA’s targeting Rab10. Cells were then transfected with SopD(R312A) to examine the localization of SopD without the confounding effects of its GAP activity. In comparison to control siRNA-treated cells, there was nearly a complete loss of SopD(R312A) localization to tubules in siRab10 knockdown cells (Fig. 1f, g). However, SopD retained localization to intracellular punctate structures, suggesting that Rab10 is required for SopD localization to tubules, but does not affect membrane binding by SopD in general. These findings also suggest a role for Rab10 in generating tubules, consistent with prior findings^27,28^.

### SopB and SopD affect Rab10 localization kinetics at *S*. Typhimurium invasion sites

To further understand the impact of SopD on Rab10, we examined the localization of this GTPase during *S*. Typhimurium infection. It is worth noting that SopD is an effector of the SPI-1 encoded T3SS and promotes invasion of host cells^16,29^. In contrast, SopD2 is primarily an effector of the SPI-2 encoded T3SS, is poorly expressed during the early stages of *S*. Typhimurium infection and is targeted to late endocytic compartments (which lack Rab10) upon translocation into host cells^19^. Taken together with our findings that SopD (but not SopD2) can disrupt Rab10 tubules in uninfected cells, we decided to focus on the early stages of *S*. Typhimurium infection.

**Fig. 2:**
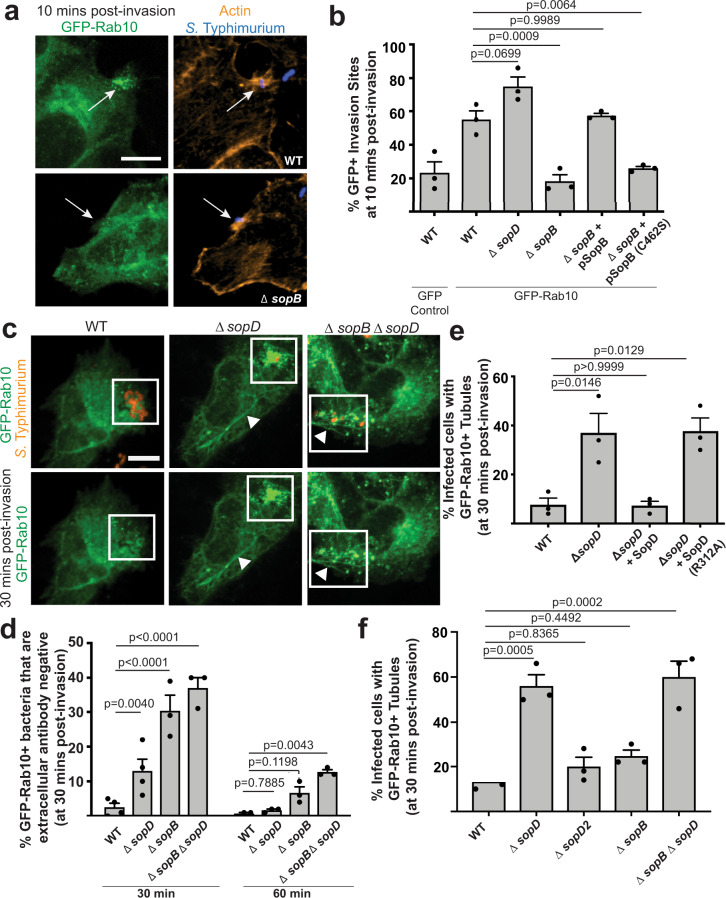
SopB and SopD affect Rab10 localization kinetics at *S*. Typhimurium invasion sites. **a** Henle 407 cells were transfected with GFP-Rab10 and infected with WT and mutant strains of *S*. Typhimurium SL1344. Cells were fixed and imaged at 10 min post-infection, actin and *S*. Typhimurium staining was used to identify invasion sites. Representative images of WT and Δ*sopB* mutant *S*. Typhimurium invasion sites. Arrows indicate invasion sites. Scale Bar, 12 μm. Data are representative of three independent experiments. **b** Quantification of GFP-Rab10 recruitment from (**a**) to actin-enriched invasion sites. In three independent experiments at least 50 invasion sites were scored for the presence or absence of GFP-Rab10 as described in the Methods Section. Data are means ± S.E.M. Statistical analysis was performed with a one-way ANOVA. **c** Representative images of Henle 407 cells transfected with GFP-Rab10 and infected with WT, Δ*sopD*, and Δ*sopB*Δ*sopD* mutant strains of *Salmonella* for 30 min. Inset shows invasion site and arrowhead denotes a Rab10^+^ tubule. Scale Bar, 12 μm. Data are representative of three independent experiments. **d** Quantification of GFP-Rab10 recruitment to bacteria from (**c**). *Salmonella* antibody staining before and after cell permeabilization was used to distinguish bacteria populations undergoing internalization, as described in the “Methods” section. In three independent experiments, bacteria (shown in red) that were inaccessible to extracellular antibody were scored for GFP-Rab10 localization in at least 50 invasion sites. Data are means ± S.E.M. Statistical analysis was performed with one-way ANOVA. **e**, **f** Quantification of the number of infected cells with GFP-Rab10^+^ tubules at 30 min post-infection following infection by the indicated strains. In three independent experiments at least 50 infected cells were scored for the presence or absence of GFP-Rab10^+^ tubules. Data are means ± S.E.M. Statistical analysis was performed with a one-way ANOVA. SCV: *Salmonella*-containing vacuole; GFP: green fluorescent protein. Source data are included in source data file.

SopD localizes to *S*. Typhimurium invasion sites^9^, therefore we tested whether Rab10 is also localized to these sites. Henle 407 cells were transfected with GFP-Rab10 and infected for 10 min with *S*. Typhimurium strains (Fig. 2a, b). Using WT *S*. Typhimurium, we observed that GFP-Rab10 localized to ~ 60% of invasion sites at 10 min post-infection (p.i.). Enrichment of Rab10 at WT *S*. Typhimurium invasion sites at this timepoint was confirmed using an antibody targeting endogenous Rab10 (Supplementary Fig. 5a). We did not observe a significant difference in GFP-Rab10 recruitment during infection with a Δ*sopD* mutant (deleted for the *sopD* gene) (Fig. 2b), suggesting that SopD is not involved in Rab10 recruitment to *S*. Typhimurium invasion sites at 10 min p.i.

SopB mediates targeting of many host-signalling factors to the invasion site via its phosphatase activity^30^. Therefore, we tested whether SopB promotes recruitment of Rab10 as well. The *S*. Typhimurium mutant strain lacking SopB (Δ*sopB*, deleted for the *sopB* gene) had a significant defect in Rab10 recruitment to the invasion site relative to WT 10 min p.i. (Fig. 2a, b), but these bacteria eventually colocalized with Rab10 by 30 min p.i. (Fig. 2d, Supplementary Fig. 6). Complementation of the Δ*sopB* bacteria with WT SopB, but not the catalytically inactive C462S mutant^31^, expressed from a low copy plasmid restored recruitment of GFP-Rab10 to invasion sites at 10 min p.i. (Fig. 2a, b). These data suggest that Rab10 localization kinetics at *S*. Typhimurium invasion sites is delayed in the absence of SopB’s lipid phosphatase activity.

At later stages of invasion (30–60 min p.i.), *S*. Typhimurium is localized to invaginated regions of the plasma membrane that subsequently undergo scission to generate SCVs containing the bacteria. These later stages of infection are identified as bacteria that become inaccessible to antibodies present in the extracellular medium (see model, Supplementary Fig. 7). We examined the localization of Rab10 to these bacteria during infection. We observed that Rab10 rarely colocalized with WT bacteria at 30 and 60 min p.i. (Fig. 2c, d, Supplementary Fig. 6). Loss of Rab10 from WT *S*. Typhimurium invasion sites at this timepoint was confirmed using an antibody targeting endogenous Rab10 (Supplementary Fig. 5b). In contrast, at 30 min p.i., Δ*sopD* mutant bacteria displayed significant colocalization with Rab10 (Fig. 2c, d, Supplementary Fig. 5b). We also observed a robust aggregation of GFP-Rab10^+^ compartments at the invasion site (not directly colocalized with bacteria) of Δ*sopD* mutant bacteria (Fig. 2c, Supplementary Fig. 8). This aggregation phenotype, at 30 min p.i., was reversed by complementation of the Δ*sopD* mutant bacteria with WT SopD but not by expression of the SopD(R312A) (Supplementary Fig. 8). Our data suggest that the GAP activity of SopD mediates the removal of Rab10 from *S*. Typhimurium invasion sites at later stages of bacterial uptake. Our findings are consistent with previous observations demonstrating that Rab GTPases reversibly associate with membranes in a manner regulated by their cycle of GTP binding and hydrolysis^32^.

A double mutant lacking both effectors (Δ*sopB*Δ*sopD*) had the greatest impairment in Rab10 localization kinetics compared to WT infection, displaying significant enrichment of Rab10 on nascent SCVs up to 60 min p.i. (Fig. 2c, d, Supplementary Fig. 6). We conclude that both SopB and SopD activity affect Rab10 localization kinetics at the invasion site, which results in timely acquisition and removal of Rab10 during invasion.

### SopD disrupts Rab10^+^ tubules during infection

In cells infected with Δ*sopD* mutant bacteria, we observed frequent localization of GFP-Rab10 to tubules (arrowheads in Fig. 2c). Consistent with our findings in non-infected cells, complementation of the Δ*sopD* mutant with WT SopD expressed by bacteria on a low copy plasmid was sufficient to induce loss of Rab10 localization to tubules during infection (Fig. 2e). In contrast, the expression of SopD(R312A) in Δ*sopD* mutant bacteria was not sufficient to disrupt Rab10 localization to tubules. Furthermore, we observed colocalization of SopD(R312A)-GFP (transfected into cells prior to infection) with TD-Rab10 on tubules and Δ*sopD* mutant *S*. Typhimurium positive compartments at 30 min p.i., unlike cells transfected with WT SopD-GFP (Supplementary Fig. 9).

This tubule phenotype was specific to infection by Δ*sopD* mutant bacteria and was rarely observed in cells infected by either Δ*sopB* or Δ*sopD2* mutant bacteria (Fig. 2f, Supplementary Fig. 6). These tubules were also detected when cells were stained using an antibody targeting endogenous Rab10 (Supplementary Fig. 5b). Collectively, our findings indicate that SopD GAP activity is required to disrupt Rab10 localization to tubules during infection.

### SopD inhibits Rab10 to promote plasma membrane scission during *S*. Typhimurium invasion

Our findings revealed that SopD modulates Rab10 function during the invasion. This suggested the possibility that the GAP activity of SopD may contribute to an established SopD phenotype. SopD is known to act cooperatively with SopB to promote scission of the plasma membrane during *S*. Typhimurium invasion^9,10^. Cells infected with Δ*sopB* or Δ*sopD* mutant bacteria exhibit a significant defect in plasma membrane scission that is readily apparent at 20 min p.i^9^. SopB promotes scission by dephosphorylation of PI(4,5)P_2_^10^. However, the mechanism(s) used by SopD to promote membrane scission during *S*. Typhimurium invasion has remained unclear. Based on our findings here, we hypothesized that SopD may promote scission of the plasma membrane by deactivating Rab10 at invasion sites.

To test this hypothesis, we used a plasma membrane scission assay developed by Terebiznik et al.^10^. Sealing was determined by measuring the accessibility of an extracellular membrane impermeant dye to the compartments forming at the base of the invasion ruffles (see model, Supplementary Fig. 7). This includes invaginations of the plasma membrane and unsealed SCVs, which were visualized using a fluorescent protein targeted to the plasma membrane (PM-mCherry) via the myristoylation and palmitoylation sequences from Lyn tyrosine kinase^33^. For our studies, we used CellMask, an amphipathic molecule that has both a lipophilic moiety for membrane insertion and a negatively charged hydrophilic dye for attachment of the probe in the plasma membrane. CellMask was added to the medium at 20 min p.i. to label the cell surface and accessible membranes prior to fixation. The degree of scission is represented as a Fluorescence Area Ratio (CellMask fluorescence area/PM-mCherry fluorescence area).

**Fig. 3:**
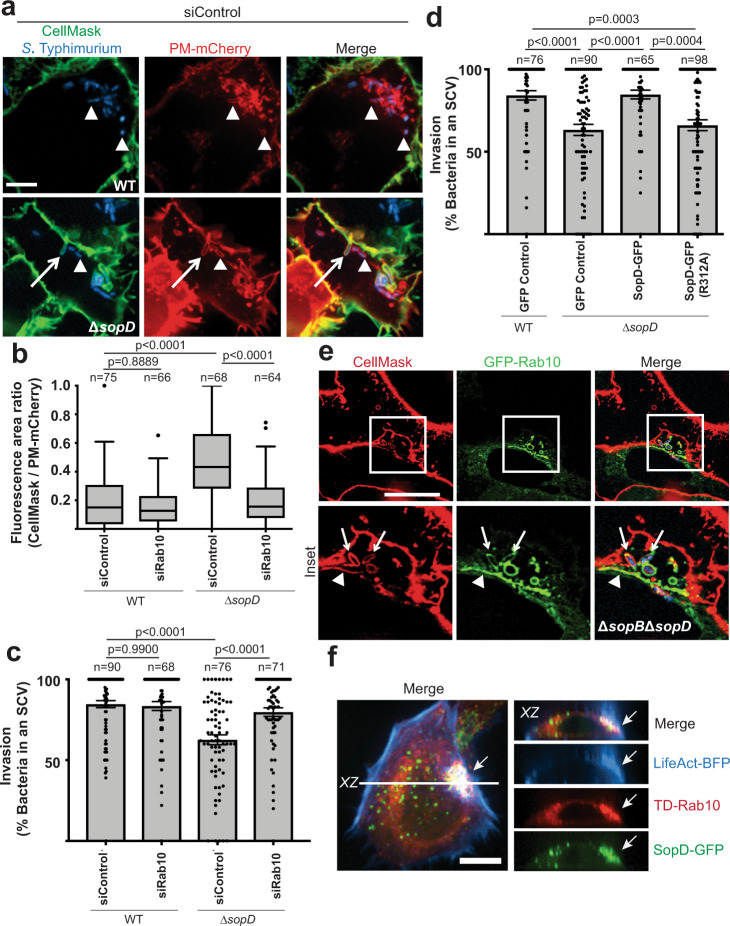
SopD inhibits Rab10 to promote plasma membrane scission during *S*. Typhimurium invasion. Henle 407 cells were transfected with the indicated siRNA and then PM-mCherry 24 h later. **a** Representative images of cells that were then infected with BFP expressing WT or *ΔsopD* mutant *Salmonella* for 20 min and labelled with CellMask (green). Arrow indicates a PM-mCherry positive *Salmonella*-containing compartment that is accessible to CellMask. Arrowhead indicates a sealed *Salmonella*-containing vacuole that is inaccessible to CellMask. Scale bar, 7 μm. Data are representative of three independent experiments. **b** The extent of membrane scission in cells transfected with Control siRNA or Rab10 siRNA #1 (see methods), followed by transfection with the indicated GFP construct and PM-mCherry 16-18 h prior to infection with WT or *ΔsopD* mutant *Salmonella*. The fluorescence area labelled with CellMask (at 20 min post-infection) compared to that labelled with PM-mCherry is used as an index of sealing at the invasion site. Data are visualized with Tukey-style boxplots, where the boxes represent the 25th, 50th/median and 75th percentiles. The whiskers denote 1.5× the IQR (interquartile range) from the median. Points denote outliers beyond 1.5× IQR. Statistical analysis was performed with one-way ANOVA. *P*-values and the number of independent cells used for measurements (*n*) are indicated in the figure. **c** Invasion represented as the percent of bacteria in *Salmonella*-containing vacuoles. The total number of bacteria that were positive for PM-mCherry at the invasion site was quantified and the proportion of these bacteria that were negative for CellMask was determined. These bacteria were in a sealed compartment that was considered an SCV and the number was used to represent the invasion efficiency. Data are means ± S.E.M. Statistical analysis was performed with one-way ANOVA. *P*-values and the number of independent cells used for measurements (*n*) are indicated in the figure. **d** Henle 407 cells were transfected with the indicated GFP construct and PM-mCherry 16–18 h prior to infection with WT and *ΔsopD* mutant *Salmonella*. Invasion represented as the percent of bacteria in *Salmonella*-containing vacuoles (as described for (c)). Data are means ± S.E.M. Statistical analysis was performed with one-way ANOVA. *P*-values and the number of independent cells used for measurements (*n*) are indicated in the figure. **e** Henle 407 cells were transfected with GFP-Rab10 and then infected with a Δ*sopB*Δ*sopD* mutant strain of *Salmonella* for 20 min and labelled with CellMask (red). Scale Bar, 21μm. GFP-Rab10 colocalizes with CellMask positive *Salmonella*-containing compartments (indicated with arrows) and CellMask positive tubules (arrowhead). Data are representative of three independent experiments. **f** Representative z-slice image of Henle 407 cells that were transfected with TD-Rab10, SopD-GFP and LifeAct-BFP, then infected with WT *S*. Typhimurium SL1344. Cells were imaged live at 10 min post-infection and actin-enriched ruffling was used to identify the invasion site. Arrow indicates site of invasion and line indicates location of slice on merged image. Scale Bar, 10 μm. Data are representative of three independent experiments. SCV *Salmonella*-containing vacuole, WT wild type, BFP blue fluorescent protein, GFP green fluorescent protein, TD TdTomato. Source data are included in source data file.

Cells infected with Δ*sopD* mutant bacteria were defective in plasma membrane scission at the *S*. Typhimurium invasion site (Fig. 3a, b), consistent with prior observations^9^. Remarkably, siRNA-mediated knockdown of Rab10 expression was sufficient to complement the plasma membrane scission defect in cells infected with the Δ*sopD* mutant bacteria. Knockdown of Rab10 was also sufficient to complement the invasion defect of Δ*sopD* mutant bacteria, which was quantified by measuring the generation of sealed SCVs (PM-mCherry^+^ bacteria inaccessible to CellMask) (Fig. 3c). Thus, in the absence of SopD, Rab10 expression restricts plasma membrane scission.

Consistent with these data, infection of cells with Δ*sopD* mutant bacteria was complemented *in trans* by transfection of host cells with SopD-GFP, as determined by measurement of sealed SCVs (Fig. 3d) and scission of the plasma membrane (Supplementary Fig. 10). In contrast, the SopD GAP mutant (SopD(R312A)-GFP) could not complement the membrane scission and invasion defect of Δ*sopD* mutant bacteria. We conclude that the GAP activity of SopD is required for its role in promoting plasma membrane scission and the generation of SCVs during *S*. Typhimurium invasion of host cells.

Our IP-MS data suggested that SopD also interacts with Rab8A, though lower spectral counts were observed in comparison to Rab10 (Supplementary Data 1, Supplementary Fig. 1). Rab8A was found to colocalize with Rab10 on tubules (Supplementary Fig. 11a) and Rab8A localization to tubules was disrupted upon co-transfection with SopD-RFP (Supplementary Fig. 11b). Consistent with this, we observed that SopD exhibits GAP activity toward Rab8A in a manner dependent on its catalytic arginine 312 residue (Supplementary Fig. 11c). However, SopD(R312A) localized to tubular compartments in a manner that depended on Rab10 whereas Rab8A had only a minor role (Supplementary Fig. 11d). Furthermore, Rab8A knockdown (confirmed by qRT-PCR) does not complement the membrane scission defect of Δ*sopD* mutant bacteria (Supplementary Fig. 11e, f). Collectively, these data suggest that Rab10 is the relevant target of SopD during *S*. Typhimurium invasion.

### Rab10 localizes to invaginated regions of the plasma membrane during host cell invasion

Under conditions when plasma membrane scission was impaired (by infection by Δ*sopB* or Δ*sopD* mutant bacteria), we observed Rab10 retention at invasion sites. This effect was most pronounced in cells infected with the Δ*sopB*Δ*sopD* double mutant, in which membrane scission is severely delayed (Fig. 3e). Under these infection conditions, invaginated plasma membrane was labeled with CellMask, as above. Notably, ~85% of GFP-Rab10^+^ bacteria were in compartments accessible to CellMask at 20 min p.i. These findings showed that Rab10 localized to invaginated portions of the plasma membrane, including tubular invaginations and nascent SCV’s, prior to scission induced by SopB and SopD.

Consistent with the findings above, SopD and Rab10 colocalized at actin-enriched invasion sites, denoted by the marker of F-actin, Lifeact-BFP^34^, at 10 min p.i. (Fig. 3f). As shown in the *XZ* cross-section, SopD-GFP and TD-Rab10 were enriched at the base of the *S*. Typhimurium invasion sites, where the plasma membrane invaginates to form nascent SCVs. Notably, SopD and Rab10 were absent from the actin-enriched protruding ‘ruffles’ that extended dorsally from the cell surface. Thus, SopD localized to sites of Rab10 recruitment early during infection, prior to the release of Rab10 from these sites at later stages of infection.

### Rab10 effectors limit scission of the plasma membrane during invasion by Δ*sopD* mutant *S*. Typhimurium

**Fig. 4:**
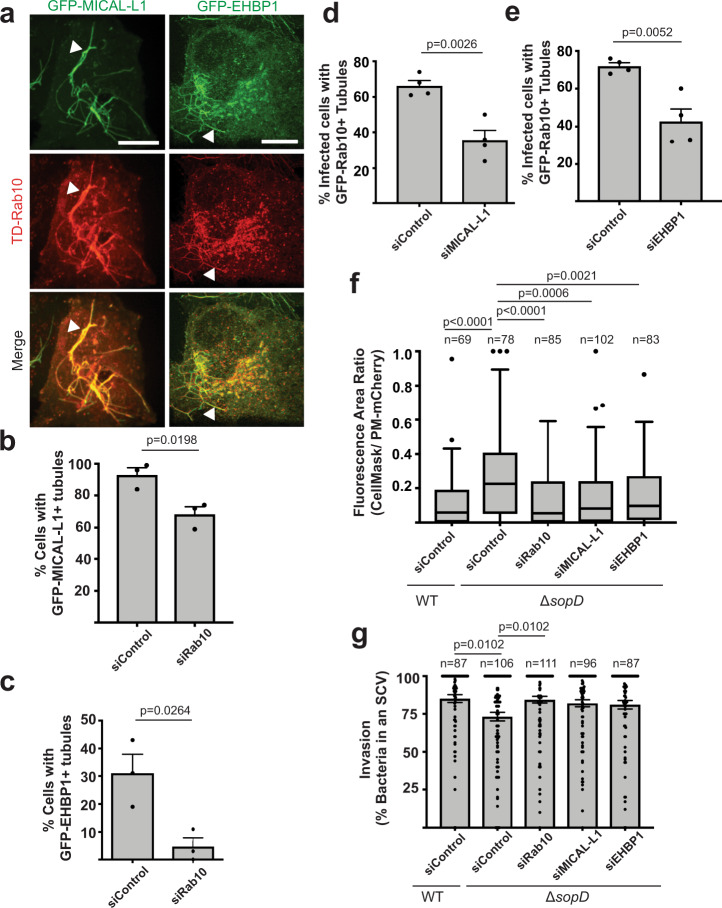
Rab10 effectors limit scission of the plasma membrane during invasion by Δ*sopD* mutant *S*. Typhimurium. **a** Representative images of Henle 407 live cells co-transfected with TD-Rab10 and GFP-MICAL-L1 or GFP-EHBP1. Arrowheads indicate colocalization on tubules. Scale Bar, 10 μm. Data are representative of three independent experiments. **b**, **c** Quantification of the presence or absence of (**b**) GFP-MICAL-L1 or (**c**) GFP-EHBP1 tubules under siControl or siRab10 knockdown conditions. At least 100 cells were quantified in three independent experiments. Data are means ± S.E.M. *P* values determined using a two-tailed unpaired Student’s *t* test. **d**, **e** Henle 407 cells were transfected with the indicated siRNA and transfected 24 h later with GFP-Rab10, then infected with Δ*sopD* mutant *S*. Typhimurium and fixed 30 min post-infection. In three independent experiments at least 50 infected cells were quantified for the presence or absence of GFP-Rab10^+^ tubules. Data are means ± S.E.M. *P* values determined using a two-tailed unpaired Student’s *t* test. **f** Henle 407 cells were transfected with the indicated siRNA (Rab10 siRNA #2, see “Methods”) and then PM-mCherry 24 h later. Cells were then infected with BFP expressing WT or *ΔsopD* mutant *Salmonella* for 20 mins and labelled with CellMask. The fluorescence area labelled with CellMask compared to that labelled with PM-mCherry is used as an index of sealing at the invasion site. Data are visualized with Tukey-style boxplots, where the boxes represent the 25th, 50th/median and 75th percentiles. The whiskers denote 1.5x the IQR (interquartile range) from the median. Points denote outliers beyond 1.5 x IQR. Statistical analysis was performed with one-way ANOVA. *P*-values and the number of independent cells used for measurements (*n*) are indicated in the figure. **g** Invasion represented as the percent of bacteria in *Salmonella*-containing vacuoles. The total number of bacteria that were positive for PM-mCherry at the invasion site was quantified and the proportion of these bacteria that were negative for CellMask was determined. These bacteria were in a sealed compartment that was considered an SCV and the number was used to represent the invasion efficiency. Data are means ± S.E.M. Statistical analysis was performed with one-way ANOVA. *P*-values and the number of independent cells used for measurements (*n*) are indicated in the figure. SCV *Salmonella*-containing vacuole, GFP green fluorescent protein, TD TdTomato. Source data are included in source data file.

Our findings above indicated that Rab10 limits plasma membrane scission during invasion with *S*. Typhimurium mutants lacking SopD (Fig. 3). Therefore, we wanted to examine how Rab10 activity could limit plasma membrane invaginations (including nascent SCV’s and other tubular invaginations of the plasma membrane) from undergoing normal scission. Since Rab GTPases usually regulate membrane trafficking events through the function of their effectors^35^, we hypothesized that known Rab10 effectors may be involved. The Rab10 effectors, MICAL-L1 (Molecule Interacting with CasL-like protein 1) and EHBP1 (EH domain-binding protein 1) are known to localize to tubular membranes^36,37^ where MICAL-L1 has been described as a signalling hub connecting a network of GTPases^37^, and EHBP1 has been shown to function as an adaptor protein capable of binding PI(4,5)P_2_ and filamentous actin^38^. Consistent with prior studies, MICAL-L1 and EHBP1 colocalized with Rab10^+^ tubules when co-transfected in epithelial cells (Fig. 4a). Furthermore, knockdown of Rab10 disrupted the frequency of MICAL-L1 and EHBP1 localization to tubules (Fig. 4b, c). Next, we tested the role of MICAL-L1 and EHBP1 during *S*. Typhimurium infection. Knockdown of either Rab10 effector (confirmed by qRT-PCR, Supplementary Fig. 12) reduced the number of infected cells with GFP-Rab10^+^ positive tubules during Δ*sopD* mutant infection (Fig. 4d, e). Thus, the Rab10 effectors MICAL-L1 and EHBP1 impact Rab10^+^ tubules during infection by Δ*sopD* mutant bacteria.

Next, we examined the role of Rab10 effectors in limiting plasma membrane scission during infection by Δ*sopD* mutant bacteria. We observed that MICAL-L1 and EHBP1 knockdown could complement the plasma membrane scission defect at Δ*sopD* mutant invasion sites to levels similar to cells with Rab10 knockdown (Fig. 4f). Knockdown of MICAL-L1 and EHBP1 also promoted Δ*sopD* mutant entry into sealed SCVs (Fig. 4g), though this effect was not statistically significant, indicating that Rab10 effectors may act cooperatively to restrict plasma membrane scission. Consistent with our findings, MICAL-L1 and EHBP1 colocalize with Rab10 at Δ*sopD* and Δ*sopB*Δ*sopD* mutant bacteria invasion sites at 20 min p.i., suggesting that these Rab10 effectors act locally on invaginations of the plasma membrane to limit scission (Supplementary Fig. 13).

### Rab10 and its effectors inhibit recruitment of Dynamin-2 during invasion by Δ*sopD* mutant *S*. Typhimurium

Although our findings revealed that Rab10 and its effectors limit plasma membrane scission during *S*. Typhimuirum invasion, we wanted to address how the inactivation of Rab10 promotes scission. Dynamin-2, a large monomeric GTPase, is recruited to *S*. Typhimurium invasion sites where it is known to play a significant role in promoting plasma membrane scission and the generation of SCVs^39^. Based on our findings here, we hypothesized that SopD-mediated inhibition of Rab10 may impact the ability of Dynamin-2 to promote SCV generation.

**Fig. 5:**
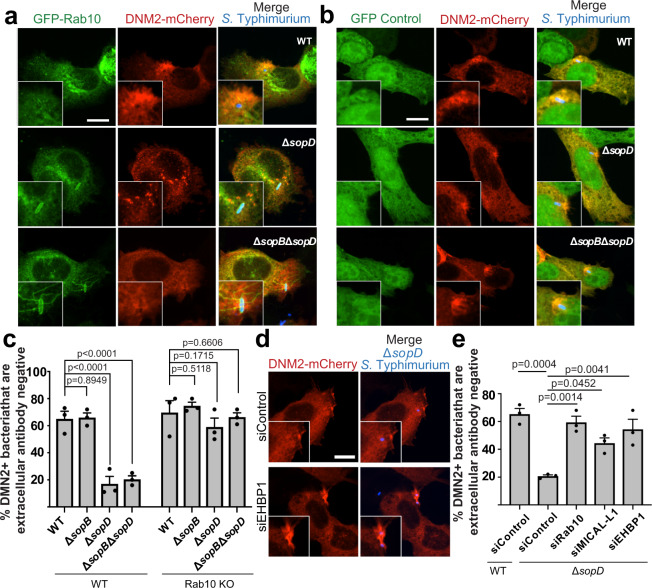
Rab10 and its effectors inhibit recruitment of Dynamin-2 during invasion by Δ*sopD* mutant *S*. Typhimurium. **a** Representative image of WT Henle 407 cells that were transfected with GFP-Rab10, and DNM2-mCherry then infected with WT, Δ*sopD*, or Δ*sopB*Δ*sopD S*. Typhimurium SL1344 for 30 min. Inset shows invasion site. Scale Bar, 13 μm. Data are representative of three independent experiments. **b** Representative image of Rab10 KO Henle 407 cells that were transfected with GFP-Rab10, and DNM2-mCherry then infected with WT, Δ*sopD*, or Δ*sopB*Δ*sopD S*. Typhimurium SL1344 for 30 min. Inset shows invasion site. Scale Bar, 13 μm. Data are representative of three independent experiments. **c** Quantification of DNM2-mCherry recruitment from (**a** and **b**). *Salmonella* antibody staining before and after cell permeabilization was used to distinguish bacteria populations undergoing internalization, as described in the “Methods” section. In at least three independent experiments, bacteria (shown in blue) that were inaccessible to extracellular antibody were scored for DNM2-mCherry localization in at least 50 invasion sites. Data are means ± S.E.M., from three independent experiments. Statistical analysis was performed with one-way ANOVA. **d** Representative image of Henle 407 cells that were transfected with the indicated siRNA and then DNM2-mCherry 24 h later. The cells were infected with Δ*sopD S*. Typhimurium SL1344 for 30 min. Inset shows invasion site. Scale Bar, 13 μm. Data are representative of three independent experiments. **e** Quantification of DNM2-mCherry recruitment at 30 min p.i. *Salmonella* antibody staining before and after cell permeabilization was used to distinguish bacteria populations undergoing internalization, as described in the Methods section. In at least three independent experiments, bacteria (shown in blue) that were inaccessible to extracellular antibody were scored for DNM2-mCherry localization in at least 50 invasion sites. Data are means ± S.E.M., from three independent experiments. Statistical analysis was performed with one-way ANOVA. WT wild type, GFP green fluorescent protein, KO knockout. Source data are included in source data file.

To test this hypothesis, we examined Dynamin-2 recruitment to *S*. Typhimurium invasion sites. Cells were transfected with Dynamin-2 fused to mCherry (DNM2-mCherry) prior to infection. We observed robust recruitment of DNM2-mCherry to sites of invasion by WT bacteria (Fig. 5a), consistent with prior observations^39^. However, robust recruitment of DNM2-mCherry was not observed in cells during Δ*sopD* mutant infection (Fig. 5a, c). Similar results were observed with Δ*sopB*Δ*sopD* mutant bacteria. In contrast, the single Δ*sopB* mutant did not display a defect in DNM2-mCherry recruitment to invasion sites. These findings indicate that SopD plays an important role in the recruitment of Dynamin-2 to *S*. Typhimurium invasion sites.

Next, we examined the role of Rab10 in Dynamin-2 recruitment to *S*. Typhimurium invasion sites. CRISPR-mediated knockout of Rab10 in Henle 407 cells (confirmed by Western blot, Supplementary Fig. 4d) did not affect recruitment of DNM2-mCherry to sites of invasion by WT bacteria or those lacking the effector SopB (Fig. 5b, c). However, knockout of Rab10 was sufficient to restore DNM2-mCherry recruitment to invasion sites by Δ*sopD* mutant bacteria. Thus, Rab10 impairs Dynamin-2 recruitment to *S*. Typhimurium invasion sites in the absence of SopD.

We also observed that MICAL-L1 and EHBP1 knockdown could complement the recruitment defect of DNM2-mCherry at Δ*sopD* mutant invasion sites (Fig. 5d, e). These findings indicate that Rab10’s effectors play an important role in modulating Dynamin-2 activity at the plasma membrane. In summary, our findings reveal that SopD-mediated inhibition of Rab10 regulates Dynamin-2 recruitment to sites of invasion.

### SopD-mediated inhibition of Rab10 promotes plasma membrane scission during *S*. Typhimurium invasion via Dynamin-2

**Fig. 6:**
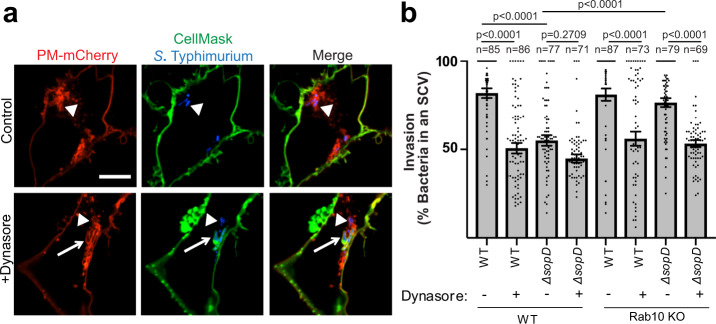
SopD-mediated inhibition of Rab10 promotes plasma membrane scission during *S*. Typhimurium invasion via Dynamin-2. **a** Representative images of WT Henle 407 cells transfected with PM-mCherry and then infected with WT BFP-*S*. Typhimurium SL1344 for 20 min and labelled with CellMask (green), with or without Dynasore treatment. Arrow indicates a PM-mCherry positive *Salmonella*-containing compartment that is accessible to CellMask. Arrowhead indicates a sealed *Salmonella*-containing vacuole that is inaccessible to CellMask. Scale Bar, 10 μm. Data are representative of three independent experiments. **b** WT or Rab10 KO Henle 407 cells were transfected with PM-mCherry 16–18 h prior to infection with WT or *ΔsopD* mutant *S*. Typhimurium with or without Dynasore treatment. Cells were then labelled with CellMask at 20 min post-infection and fixed. Invasion is represented as the percent of bacteria in SCVs. The total number of bacteria that were positive for PM-mCherry at the invasion site was quantified and the proportion of these bacteria that were negative for CellMask was determined. These bacteria were in a sealed compartment that was considered an SCV and the number was used to represent the invasion efficiency, at 20 min p.i. Data are means ± S.E.M. Statistical analysis was performed with one-way ANOVA. *P*-values and the number of independent cells used for measurements (*n*) are indicated in the figure. SCV *Salmonella*-containing vacuole, WT wild type, BFP blue fluorescent protein, GFP green fluorescent protein, KO knockout. Source data are included in source data file.

Our findings suggested that SopD promotes plasma membrane scission and subsequent *S*. Typhimurium invasion via Dynamin-2 activity. We tested this possibility by performing a plasma membrane scission assay in the presence or absence of Dynasore, a potent inhibitor of Dynamin-2^40^. As expected, Dynasore treatment significantly reduced the invasion of WT bacteria (Fig. 6a, b)^39^. Invasion with Δ*sopD* mutant bacteria was impaired in comparison to WT *S*. Typhimurium invasion. However, invasion of Δ*sopD* mutant bacteria was not significantly affected by Dynasore treatment, consistent with defective Dynamin-2 recruitment to invasion sites (Fig. 5). Invasion of Δ*sopD* mutant bacteria was complemented to nearly WT levels in a Rab10 KO Henle 407 cell line, consistent with our siRab10 knockdown study. However, this complementation was lost when Dynamin-2 was inhibited. Our findings reveal how SopD-mediated inhibition of Rab10 can promote bacterial invasion through enabling the recruitment and subsequent activity of Dynamin-2.

## Discussion

Our study identifies Rab10 as a host cell target of SopD. Furthermore, we demonstrate that SopD has a direct GAP activity towards Rab10 in vitro and modulates Rab10 localization during *S*. Typhimurium invasion. Our findings are consistent with prior studies indicating that the related effector SopD2 can inhibit the activity of multiple Rab GTPases^20–22^. We also show that the catalytic arginine residue required for the GAP activity of SopD2^21^ is present in its paralog SopD and necessary for its GAP activity towards Rab10. SopD2 was also shown to bind and inactivate Rab10 in vitro^21^. However, the significance of this activity during infection was not determined.

**Fig. 7:**
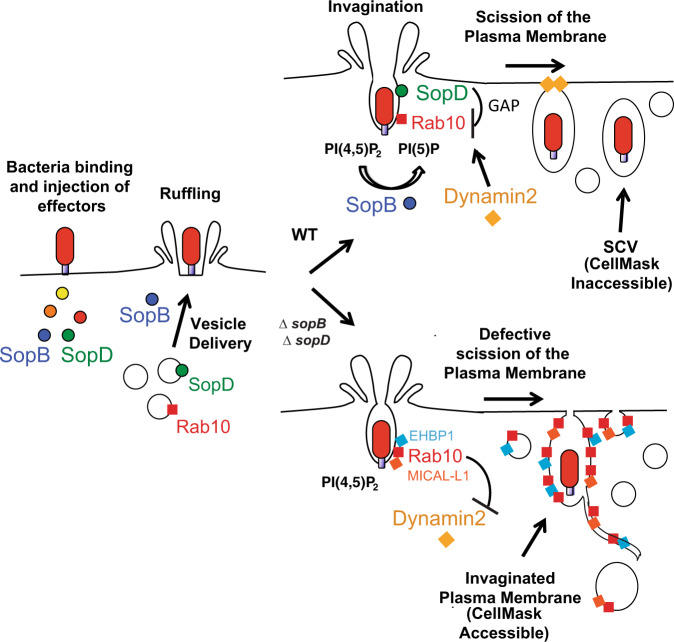
The *Salmonella* effector SopD targets Rab10 to promote plasma membrane scission during invasion. Model depicting the results of this study. *Salmonella* use a type 3 secretion system (T3SS) to inject virulence factors called effectors into host cells. These effectors induce cytoskeletal rearrangements and changes in phosphoinositide dynamics in order to form an invasion-associated ruffle. We demonstrate that the *Salmonella* lipid phosphatase, SopB, promotes the recruitment of Rab10 to the *S*. Typhimurium invasion site, as previously shown for SopD^9^. SopD and Rab10 colocalize at the base of the *Salmonella* invasion ruffle where the plasma membrane invaginates to form a *Salmonella*-containing vacuole. Together SopB and SopD contribute in Rab10 removal from the forming vacuole and promote plasma membrane scission at the invasion site. SopB and SopD contribute to vacuole scission through PI(4,5)P_2_ hydrolysis and Rab10 GAP activity, respectively. SopD-mediated inactivation of Rab10 leads to the recruitment of Dynamin-2 and subsequent scission of plasma membrane to form an SCV. In the absence of SopB and SopD, vacuole scission is defective and Rab10^+^ tubules (including tubular invaginations of the plasma membrane and tubular endosomes) are prominent at the invasion site. SCV: *Salmonella*-containing vacuole; WT: wild type.

Here, we show that SopD-mediated inhibition of Rab10 contributes to *S*. Typhimurium invasion of host cells by promoting scission of the plasma membrane at bacterial invasion sites (see model in Fig. 7). During the early stages of invasion, Rab10 localizes to invaginated portions of the plasma membrane. Consistent with these findings, Rab10 has been observed on phagocytic cups prior to their closure^41^, although its role in the sealing of phagosomes has been unclear. We find that inhibition of Rab10 function, either through the GAP activity of SopD or through knockdown of Rab10 expression, promotes membrane scission and internalization of bacteria into SCVs. To our knowledge, this is the first study to show that direct inactivation of a Rab family GTPase promotes bacterial invasion.

SopD2 is primarily an effector of the SPI-2 encoded T3SS, poorly expressed during the early stages of *S*. Typhimurium infection and unlikely able to promote bacterial invasion through its ability to inhibit Rab10. Indeed, we show that SopD (Fig. 1d, e; Fig. 2c, e), but not SopD2 (Supplementary Figs. 3, 6), is sufficient to disrupt Rab10^+^ tubule localization in transfected cells and during infection. However, *S*. Typhimurium expresses both SopD and SopD2 after entry into SCVs and both effectors are required for optimal bacterial growth in macrophages^18^. Thus, it will be important to determine whether inhibition of Rab10 by SopD and/or SopD2 impacts the later stages of infection of host cells.

Our IP-MS data revealed that SopD also interacts with Rab8A. However, Rab8A knockdown does not complement the membrane scission defect of Δ*sopD* mutant bacteria (Supplementary Fig. 11e). These data suggest that Rab10 is the relevant target of SopD during *S*. Typhimurium invasion. In future studies, it will be important to determine whether inhibition of Rab8A by SopD (and/or SopD2^21^) impacts other pathways or stages of infection. Of note, during the review of this manuscript Galan and colleagues revealed that SopD can target Rab8 to positively and negatively modulate the inflammatory response^42^.

Our findings highlight the remarkable cooperativity of SopB and SopD to promote bacterial invasion. SopB has lipid phosphatase activity that can promote plasma membrane scission through dephosphorylation of PI(4,5)P_2_ to generate PI(5)P^11,12^ and we find that SopB contributes to the dynamic modulation of Rab10 localization. First, SopB promotes membrane reorganization at invasion sites, which promotes recruitment of Rab10 and SopD, along with many other signalling molecules through molecular mechanisms that remain unclear^9,30^. We determined that Δ*sopB* mutant bacteria eventually acquire Rab10 and are also delayed in its removal from nascent SCVs. This could be explained by SopB-mediated dephosphorylation of PI(4,5)P_2_, which has been shown to affect the charge of the membrane surrounding the bacteria and alter the localization of many signalling molecules^43^. Mammalian phosphatidylinositol polyphosphate phosphatases, such as Synaptojanin 1, play a significant role in plasma membrane scission during endocytosis. Synaptojanin 1 was shown to mediate PI(4,5)P_2_ to PI(4)P conversion at sites of high membrane curvature induced by the BAR domain-containing protein endophilin^44^. Spatial elimination of PI(4,5)P_2_ was found to cooperate with Dynamin to initiate fission of membrane tubules. Thus, dephosphorylation of PI(4,5)P_2_ appears to be a critical step in the scission of the plasma membrane.

Our findings reveal that SopD inhibits Rab10 to promote bacterial invasion. We also show that Rab10 must be cycled off (undergo GTP hydrolysis) to promote plasma membrane scission during *S*. Typhimurium invasion via Dynamin-2. The mechanism by which Rab10 restricts plasma membrane scission during infection by Δ*sopD* mutant bacteria remains an important question. Based on our work and prior studies, we propose that several possible mechanisms are likely to be involved. First, it is known that Rab10 promotes PI(4,5)P_2_ production via an Arf6-dependent pathway^45^, and may thus counter the ability of SopB to dephosphorylate this lipid during infection. Second, several Rab10 effectors are known to bind to PI(4,5)P_2_^38,46,47^ and may act to sequester this lipid from SopB. Third, Rab10 effectors may generate a membrane microdomain that spatially restricts scission via Dynamin-2. For example, EHBP1 is known to promote tubular endosome formation by simultaneously binding to PI(4,5)P_2_ and filamentous actin^38^. MICAL-L1 may act in a similar manner since members of the MICAL family are known to modulate the actin cytoskeleton^48^. It is interesting that Rab10 was shown to bind Dynamin-2 preferentially in its GDP-bound state^49^. Therefore, under normal infection conditions SopD-mediated inactivation of Rab10 may have the dual benefit of i) releasing Rab10-mediated stabilization of plasma membrane invaginations and ii) simultaneously promoting recruitment of Dynamin-2 via its ability to bind GDP-bound Rab10. Further studies are required to determine the ability of Rab10 to stabilize plasma membrane invaginations and regulate Dynamin-2 activity.

Here we show that EHBP1 and MICAL-L1 impact Rab10^+^ tubule regulation during infection and inhibit plasma membrane scission during infection by Δ*sopD* mutant bacteria. In future studies it will be important to determine the full complement of Rab10 effectors required for the formation of these structures. It will also be important to examine how Rab10 regulates plasma membrane dynamics in other physiological contexts. Our findings provide new insight into the many roles of Rab10. Prior studies have identified a diverse array of Rab10 activities in multiple intracellular compartments, including endosomes, the endoplasmic reticulum, the Golgi/TGN, Glut4-containing exocytic vesicles, neuronal axons and primary cilia (reviewed in^50^).

Plasma membrane scission has been studied during the internalization of other pathogens such as *Yersinia pseudotuberculosis*, which is known to exploit host factors to promote plasma membrane scission during the invasion of host cells^51^. In contrast, *Toxoplasma gondii* utilizes unidentified secreted virulence proteins to promote scission via a twisting mechanism^52^. Impairment of plasma membrane scission causes swelling and death of the parasites, indicating this step is critical during infection and may be a suitable target for antimicrobial development. Whether other pathogens inhibit Rab10 activity to promote invasion of host cells, in a manner similar to *S*. Typhimurium, will be an important topic for future studies.

## Methods

### Plasmids

All primers used for plasmid generation/mutagenesis are listed in Supplementary Table 1. Gene encoding the *S*. Typhmurium effector SopD was cloned from the genomic DNA of *S*. Typhmurium SL1344 into the pcDNA5-FlagBirA^*^-FRT/TO vector^24^ with a C-terminal tag. The following primers were used for amplification of *sopD*, with engineered restriction sites AscI and NotI for cloning: KBP1 5′TATAGGCGCGCCATGCCAGTCACTTT

AAGCTTCG 3′ and KBP2 5′ TTAAGCGGCCGCTGTCAGTAATATATTACGACTGCACC 3′. WT pKH3-tdTomato-Rab10 (TD-Rab10) was previously described^46^ and was obtained from Dr. Zhen-Ge Luo. WT, CA (constitutively active) and DN (dominant negative) peGFP-Rab10 were obtained from Dr. Marci Scidmore^53^ and WT GFP-Rab8A was obtained from Dr. Amira Klip^54^. GFP-MICAL-L1 was received as a gift from Dr. Steve Caplan and was previously described^55^. GFP-EHBP1 was obtained as a gift from Dr. Mark McNiven and was described previously^56^. The 2PH-PLCδ-GFP construct was obtained as a gift from Dr. Mario J. Rebecchi and was previously described^11^. SopD-GFP was described previously^19^. SopD (R312A)-GFP was created by an inverse PCR mutagenesis strategy^57^ from SopD-GFP using the following primers: KBP3 5′ CTAATATATTACTGACGGATCCACCGGTCGC 3′ and KBP4 5′ CACTGCACCCATCTTTACCAATGTGCAAAG 3′. Primers were synthesized with 5′ phosphates. Likewise, the complementation plasmid for expression in *Salmonella*, HA-SopD-pACYC184, was mutated to generate 2HA-SopD(R312A)-pACYC184 using the following primers: KBP5 5′ CTAATATATTACTGACACTCGAGTATC

CGTATGATGTGC 3′ and KBP6 5′ CACTGCACCCATCTTTACCAATGTGCAAAG 3′. For pCMV-PM-mCherry, myristoylation and palmitoylation sequences from Lyn tyrosine kinase^33^ were fused to mCherry. The restriction sites BamHI and NotI in the peGFP-N1 vector were used for cloning (with the eGFP removed) using the primer pair, KBP7 5′ TGCAGGATCCGCCACCATGGGCTGCATTAAAAGCAAACGCAAAGATA

TGGTGAGCAAGGGCGAGGAGGATAACATG 3′ and KBP8 5′ TGCAGCGGCCGCTTACT

TGTACAGCTCGTCCATGCCGCCGGT 3′. The pET10 HIS-TEV-Rab10^58^ obtained from Dr. Roger Goody, pMCSG7 HIS-TEV-SopD, and pMCSG7 HIS-TEV-SopD_2_ were previously described^22^. For pMCSG7 HIS-TEV-SopD (R312A), pMCSG7 HIS-TEV-SopD was amplified with a R312A primer pair, KBP9 5′ GATGGGTGCAGTGCTAATA

TATTACTGAC 3′ and KBP10 5′ GTCAGTAATATATTAGCACTGCACCCATC 3′ followed by DpnI digestion. The sequence of the mutant was confirmed by Eurofins Genomics. Rab8(3-184) was constructed into pMCSG7 with the ligation independent cloning approach^59^. Rab8 gene was amplified with primer pair KBP11 5′TACTTCCAATCCAA TGCCAAGACCTACGATTACCTGTTCAAGCTG-3′ and KBP12 5′-TTATCCACTTCCA

ATGTTACCCCTGGGGGCTGTTGCCTTCC-3′. The DNM2-mCherry plasmid was purchased from Addgene (Plasmid#27689).

The construct BFP-pFPV25.1, used to generate fluorescent *Salmonella*, was created by mutagenesis of pFPV25.1^60^. The following primers were used with the inverse PCR mutagenesis approach:^57^ KBP13 5′ GGATCCTCTAGATTTAAGAAGGAGATATACATA

TGAGCGAGCTGATTAAGGAGAAC 3′ and KBP14 5′CTTGCATGCCTGCAGGAGATTTAATTAAGCTTGTGCCCCAGTTTG 3′. For this mutagenesis, the plasmid pTagBFP-C-Rab5 that was obtained as a gift from Gia Voeltz (Addgene plasmid # 49147)^61^, was used as a template to amplify BFP. Likewise, BFP-LifeAct was created from RFP-LifeAct^34^ and pTagBFP-C-Rab5 using the following primers: KBP15 5′ AGGAAGAAGGTACCGCGGGCCCGGGATCCATGAGCGAGCTGATTAAGGAGAAC 3′ and KBP16 5′ GGTATGGCTGATTATGATCAGTTATCTAGATTAATTAAGCTTGTGCCCCAGTTTG 3′.

### Cell culture

Henle 407 and HEK 293T cells were obtained from the American Type Culture Collection (ATCC). Although Henle 407 cell cultures have been shown to contain HeLa cell chromosomes, our Henle 407 cells were used between passages 5–25 and maintained a distinct morphology relative to HeLa cells. Flp-In T-REx 293 cells were obtained from Invitrogen. Cell cultures were maintained in growth medium (DMEM, high glucose (HyClone) supplemented with 10% FBS (Wisent)) at 37 °C in 5% CO_2_.

Using the Flp-In system (Invitrogen), Flp-In T-REx 293 cells stably expressing FLAGBirA* alone or SopD-FLAGBirA* were generated. After selection (DMEM + 10% FBS + 200 ug/ml Hygromycin B), 5 × 150 cm^2^ plates of sub-confluent (80%) cells were incubated for 24 h in complete media supplemented with 1 ug/ml tetracycline (Sigma) before harvesting cells.

For microscopy-based experiments, Henle 407 cells were seeded in 24-well tissue culture plates containing 1 cm coverslips at a concentration of 6 × 10^4^ cells/well 24 h or 3 × 10^4^ cells/well 48 h before use. For live cell imaging, cells were seeded in μ-Slide 8-well glass bottom chambers (ibidi) 24 h before use at a concentration of 4.0 × 10^4^ cells/well.

### Immunoprecipitation coupled to mass spectrometry (IP-MS)

SopD and control cell pellets were weighed, and 1:4 pellet weight:lysis buffer (by volume) was added to each sample. Lysis buffer consisted of 50 mM HEPES-NaOH (pH 8.0), 100 mM KCl, 2 mM EDTA, 0.1% NP40, 10% glycerol, 1 mM PMSF, 1 mM DTT and 1:500 protease inhibitor cocktail (Sigma-Aldrich, St. Louis, MO). On resuspension, cells were incubated on ice for 10 min, subjected to one additional freeze–thaw cycle, then centrifuged at 27000 × *g* for 20 min at 4 °C. Supernatant was transferred to a fresh 15 ml conical tube, and 1:1000 benzonase nuclease (Novagen) plus 30 μl packed, pre-equilibrated anti-FLAG M2 agarose beads (Sigma-Aldrich) were added. The mixture was incubated for 2 h at 4 °C with end-over-end rotation. Beads were pelleted by centrifugation at 1000 × *g* for 1 min and transferred with 1 ml of lysis buffer to a fresh centrifuge tube. Beads were washed once with 1 ml lysis buffer and twice with 1 ml ammonium bicarbonate (ammbic) rinsing buffer (50 mM ammbic, pH 8.0, 75 mM KCl). Elution was performed by incubating the beads with 150 μl of 125 mM ammonium hydroxide (pH >11). The elution step was repeated twice, and the combined eluate centrifuged at 15000 × *g* for 1 min, transferred to a fresh centrifuge tube and lyophilized. One microgram of MS-grade TPCK trypsin (Promega, Madison, WI) dissolved in 70 μl of 50 mM ammbic (pH 8.3) was added to the Flag eluate and incubated at 37 °C overnight. The sample was lyophilized and brought up in 0.1% formic acid.

### Mass spectrometry

High performance liquid chromatography was conducted using a 2 cm pre-column (Acclaim PepMap 50 mm × 100 um inner diameter (ID)), and 50 cm analytical column (Acclaim PepMap, 500 mm × 75 um diameter; C18; 2 um; 100 Å, Thermo Fisher Scientific, Waltham, MA), running a 120 min reversed-phase buffer gradient at 225 nl/min on a Proxeon EASY-nLC 1000 pump in-line with a Thermo Q-Exactive HF quadrupole-Orbitrap mass spectrometer. A parent ion scan was performed using a resolving power of 60,000, then up to the twenty most intense peaks were selected for MS/MS (minimum ion count of 1000 for activation), using higher energy collision induced dissociation (HCD) fragmentation. Dynamic exclusion was activated such that MS/MS of the same m/z (within a range of 10 ppm; exclusion list size = 500) detected twice within 5 s were excluded from analysis for 15 s. For protein identification, Thermo.RAW files were converted to the.mzXML format using Proteowizard^62^ then searched using X!Tandem^63^ and Comet^64^ against the Human RefSeq Version 45 database (containing 36113 entries). Search parameters specified a parent ion mass tolerance of 10 ppm, and an MS/MS fragment ion tolerance of 0.4 Da, with up to 2 missed cleavages allowed for trypsin. Variable modifications of + 16@M and W, +32@M and W, +42@N-terminus, and +1@N and Q were allowed. Proteins identified with an iProphet cut-off of 0.9 (corresponding to ≤1% FDR) and at least two unique peptides were analyzed with SAINT Express v.3.3. For SopD analysis, eight control runs (from cells expressing the FlagBirA* epitope tag) were collapsed to the two highest spectral counts for each prey and compared to the two technical replicates and two biological replicates of SopD anti-FLAG IP. High confidence interactors were defined as those with BFDR≤0.01.

### Transfections and RNA interference

Transfections were performed using GeneJuice (VWR Scientific) or X-treme GENE9 (Roche) according to the manufacturer’s instructions. For siRNA-mediated knockdown, cells were seeded in 24-well tissue culture plates at a concentration of 3 × 10^4^ cells/well 24 h before use. Cells were then transfected with 100 nM siRNA using Lipofectamine RNAiMax (Invitrogen) as recommended by the manufacturer for 48 h. Rab10-directed siRNAs (#1: SASI_Hs02_00348924 and #2: SASI_Hs01_00041007), as well as MICAL-L1 (SASI_Hs02_00361239), EHBP1 (SASI_Hs01_00097428) and Rab8A (SASI_Hs02_00339466) directed siRNAs were obtained from Sigma Aldrich. For control knockdown, MISSION^®^ siRNA Universal Negative Control (Sigma Aldrich, #SIC001) was used.

### CRISPR knockout

To disrupt Rab10 expression in Henle 407 cells, Rab10-specific single-guide RNA (sgRNA) was designed using the online tool http://guides.sanjanalab.org/. The sgRNA sequences were sgRNA#1 (sgRNA1-F: 5′-CACCGCATTGCGCCTCTGTAGTAGG-3′, sgRNA1-R: 5′-AAACCCTACTACAGAGGCGCAATGC-3′); sgRNA#2 (sgRNA2-F: 5′-CACCGGGATATCTTCAGCTAACGTG-3′, sgRNA2-R: 5′-AAACCACGTTAGCTGAAGATATCCC-3′); sgRNA#3 (sgRNA3-F: 5′-CACCGCTAGTATATGACATCACCAA-3′, sgRNA3-R: 5′-AAACTTGGTGATGTCATATACTAGC-3′). Custom sgRNA oligonucleotides were synthesized by Sigma Aldrich. The CRISPR/Cas9 vector pSpCas9 (BB)-2A-Puro (pX459) was obtained from Dr. Chi-Chung Hui. For ligation into the BbsI site of pX459, a CACCG sequence was added to the 5′ flanking sequences of the sense oligonucleotides and an AAAC sequence was added to the 5′ flanking sequences of the anti-sense oligonucleotides. The sgRNAs were inserted into pX459 vector. WT Henle 407 cells were transfected with the ligated vector and 48 h later the transfected cells were selected by Puromycin (2ug/ml) for another 48 h. Single cells were then transferred into a 96-well plate to grow until confluent. Knockout efficiency was determined by western blot analysis.

### Bacterial strains and infections

Infections were performed with WT *S*. Typhimurium SL1344^65^ and isogenic mutants lacking the effectors of interest. Mutants in the *S*. Typhimurium SL1344 background lacking SopD (Δ*sopD*)^18^, Δ*sopD* +pSopD^18^, SopB(Δ*sopB*)^66^, ∆*sopB* + pSopB^67^, and ∆*sopB* + pSopB C462S^66^, as well as lacking both SopB and SopD (Δ*sopB*Δ*sopD*)^9^ were described previously. The WT BFP- *S*. Typhimurium SL1344 as well as the isogenic BFP-Δ*sopD* and BFP-Δ*sopB*Δ*sopD* strains were constructed by transforming each strain with BFP-pFPV25.1, a plasmid expressing BFP under control of the *rpsM* promoter. SopD(R312A) was expressed in Δ*sopD* mutant *S*. Typhimurium by transforming the pACYC184 SopD(R312A) low copy plasmid.

A previously established approach was used for infection of epithelial cells, using late-log *S*. Typhimurium cultures as inocula^68^. Briefly, bacteria were pelleted at 10,000 × *g* for 2 min and resuspended in PBS, pH 7.2. The bacteria were diluted and added to cells at 37 °C for 10 min. For invasion experiments, bacteria were diluted 1:50 in PBS. If applicable, cells were fixed with 2.5% paraformaldehyde in PBS at 37 °C for 10 min.

### Immunofluorescence staining

For all fixed microscopy-based experiments, cells were fixed with 2.5% paraformaldehyde (PFA) in PBS for 10 min at 37 °C, unless indicated otherwise. Immunostaining was performed as previously described^69^ using the following primary antibodies: rabbit polyclonal anti-*S*. Typhimurium LPS (Difco), mouse monoclonal anti-GFP (clone 3E6, Molecular Probes), and mouse monoclonal anti-Rab10 (Sigma).

The following secondary antibodies were used in this study: Alexa Fluor 488-conjugated goat anti-mouse IgG (Molecular Probes), Alexa Fluor 568-conjugated goat anti-rabbit IgG (Molecular Probes), Alexa Fluor 350-conjugated goat anti-rabbit IgG (Molecular Probes), and Alexa Fluor 647-conjugated goat anti-rabbit IgG (Molecular Probes). AlexaFluor Phalloidin from Molecular Probes (ThermoFisher) or ActiStain Phalloidin (Cytoskeleton) was used to visualize F-actin. CellMask Deep Red Plasma membrane stain (Thermo Fisher Scientific) was used as a membrane impermeant dye to identify compartments open to the extracellular space and was used without cell permeabilization.

For the invasion site recruitment experiment, F-actin and *S*. Typhimurium staining were used to denote the site of invasion at 10 min p.i. An enrichment of the indicated protein’s signal at the *S*. Typhimurium invasion site, relative to the signal in the cytosol, was considered a positive recruitment. For vacuole recruitment experiments, immunostaining of bacteria before permeabilization and after permeabilization was used^70^. Recruitment to bacteria inaccessible to extracellular antibody was quantified. Cells were counted using a Leica DMIRE2 inverted epifluorescence microscope.

### Plasma membrane scission assay

Plasma membrane scission was measured as previously described^9,10^. Briefly, cells were grown in μ-Slide 8-well glass bottom chambers (ibidi) at a concentration of 4.0 × 10^4^ cells/well and transfected 16–18 h before invasion with PM-mCherry. For experiments conducted using siRNA knockdown, cells were seeded at a concentration of 4.0 × 10^4^ cells/well and transfected with siRNA, 24 h later growth media was changed and cells were transfected with PM-mCherry 16–18 h before invasion. Cells were infected for 10 min with BFP expressing *S*. Typhimurium (1:30), extracellular bacteria were removed by extensive washing with PBS, and cells incubated for an additional 8 min in growth media. Cells were then washed with PBS and cells were incubated for 2 mins with CellMask Deep Red Plasma membrane stain (Thermo Fisher Scientific) at a 1X working solution (5 μg/ml), as recommended by the manufacturer. Cells were then PFA fixed for 10 min and imaged immediately after by confocal microscopy. Scission was measured by outlining the area positive for extracellular CellMask compared to the area of all compartments forming at the base of the invasion ruffles, which were visualized using PM-mCherry. The degree of scission is represented as a Fluorescence Area Ratio (CellMask fluorescence area/ PM-mCherry fluorescence area).

For plasma membrane scission assays performed with Dynasore treatment, the above protocol was altered as follows: cells were pre-treated with Dynasore (80 μM, 0.4% DMSO in growth media) for 30 min prior to infection. Cells were then infected for 10 min with BFP expressing *S*. Typhimurium (1:30 in D-PBS with 80 μM Dynasore & 0.4% DMSO), extracellular bacteria were removed by extensive washing with PBS, and cells incubated for an additional 8 min in growth media (with 80 μM Dynasore and 0.4% DMSO). Cells were then washed with PBS and cells were incubated for 2 mins with CellMask. The control groups were treated with growth media or *S*. Typhimurium containing D-PBS with 0.4% DMSO in corresponding steps.

### Confocal microscopy

Unless otherwise indicated, cells were imaged using a Quorum spinning disk microscope with a ×63 oil immersion objective (Leica DMIRE2 inverted fluorescence microscope equipped with a Hamamatsu Back-Thinned EM-CCD camera or Hamamatsu CMOS FL-400 camera, spinning disk confocal scan head) and Volocity 6.3 acquisition software (Improvision)). Confocal *z*-stacks of 0.3 μm were acquired and images were analyzed with Volocity 6.3 software.

### Co-immunoprecipitations

For in vivo co-immunoprecipitation experiments, HEK 293T cells were seeded into 10 cm diameter tissue culture dishes at approximately 10% confluency and grown at 37 °C in 5% CO_2_. After 24 h cells were co-transfected with epitope-tagged plasmids harbouring the GFP-Rab10 (wild type or mutants) or a control plasmid and SopD-RFP (6 μg total DNA) for 24 hr, respectively. Cells were washed in PBS and lysed in Lysis Buffer (50 mM Tris-HCl, pH 7.4, 150 mM NaCl, 1 mM EDTA, 1% Triton X-100) supplemented with 1 mM phenylmethylsulfonyl fluoride (PMSF), 5 mM NaF, 5 mM NaVO_4_, 10 μg/ml aprotinin 10 mg/ml, 10 μg/ml leupeptin, and 1 μM pepstatin A. Cell lysates were incubated overnight with RFP-Trap^®^ resin (Chromotek, 15 μl packed volume), washed three times in Lysis Buffer, and eluted with 50 μl of 2x SDS-PAGE loading buffer. Samples were boiled for 10 min.

### Western blots

Cell lysates and proteins eluted from co-immunoprecipitations were resolved by 12% SDS-PAGE, transferred to PVDF membrane (Bio-Rad), and probed with antigen-specific primary antibodies. The following primary antibodies were used for Western Blot detection: rabbit polyclonal anti-GFP (Molecular Probes), rat monoclonal anti-RFP (clone 5F8, Chromotek), mouse monoclonal anti-Rab10 (Sigma), and mouse monoclonal (6C5) anti-GAPDH (Millipore).

Blocking was performed with 5% skim milk. For all analyses, HRP-conjugated secondary antibodies were used (peroxidase-conjugated goat anti-rabbit IgG (Sigma), peroxidase-conjugated goat anti-rat IgG (Jackson ImmunoResearch) or peroxidase-conjugated goat anti-mouse IgG (Jackson ImmunoResearch)) and detection was performed using ECL detection system (Luminata Classico Western HRP substrate (Millipore) or SuperSignal West Femto Maximum Sensitivity Substrate (Thermo)).

### Protein expression and purification of SopD, SopD(R312A), SopD2, Rab8 and Rab10

SopD and SopD(R312A) were expressed in SHuffle® T7 express lysY E.coli C3030 (NEB, Ipswich, MA), while SopD2 and Rab8 in BL21(DE3). An overnight culture was inoculated into 1 L Terrific Broth (TB) media supplemented with 100 µg/ml ampicillin. IPTG at a final concentration of 0.5 mM was used to induce protein expression once OD_600_ reached 1.0. The induced culture was incubated with shaking overnight at 20 °C when expressed in BL21(DE3) or for 4 h at 37 °C when expressed in C3030. After expression, the cell pellet was harvested, resuspended, and lysed in buffer containing 50 mM HEPES pH 8.0, 400 mM NaCl, 0.5 mM TCEP. The target protein was purified with affinity Talon resin. Afterwards, the protein was subjected to TEV cleavage to remove the His-tag while being dialyzed against a buffer containing 20 mM HEPES pH 8.0, 150 mM NaCl and 0.5 mM TCEP at 4 °C overnight. Following the cleavage, TEV protease, which is His-tagged, was removed by Ni-NTA resin. The clean protein was further purified by size exclusion chromatography using a BioRad SEC650 column running in the same buffer as that used for dialysis.

Rab10 was expressed in ArcticExpress (DE3) (Agilent Technologies). An overnight culture supplemented with 100 µg/ml ampicillin and 20 µg/ml gentamicin was inoculated into 6 L TB media supplemented with 100 µg/ml ampicillin. The protein was expressed at 12 °C overnight using 0.5 mM IPTG when OD_600_ reached 1.0. For Rab10 purification, 1 mM MgCl_2_ and 10 µM GDP was added into buffer used for SopD. Rab10 was also purified with Talon resin and applied to SEC650 using a running buffer containing 20 mM HEPES pH 8.0, 150 mM NaCl, 0.5 mM TCEP, 1 mM MgCl_2_ and 10 µM GDP.

### GAP assay

Purified SopD, SopD(R312A) and SopD2, at a concentration of 8 µM, were incubated with 8 µM Rab8 or Rab10 in a 40 µL reaction system containing 20 mM HEPES pH 8.0, 150 mM NaCl, 10 mM MgCl_2_ and 0.5 mM TCEP, at room temperature for 200 min. After the reactions, 160 µL of freshly mixed malachite green solution was added to the reactions for 15 min. The malachite green solution was mixed with 1.5 ml of 5.72% ammonium molybdate dissolved in 6 M HCl, 1.5 ml of 2.32% polyvinyl alcohol dissolved in H_2_O, 3 ml of 0.406‰ malachite green dissolved in H_2_O and 3 ml of H_2_O^71^. The concentration of free inorganic phosphate (_Pi_) produced in each reaction was used as a read out of GTP hydrolysis. The concentration of _Pi_ was calculated for each reaction using the absorbance values measured at 630 nm with a microplate reader (Molecular Devices M2^e^) and _Pi_ standard curve. The concentration of _Pi_ produced in each condition was normalized to the Rab10 only condition, after removing the background signal from SopD, SopDR312A or SopD2 alone. All the reactions were duplicated and the GAP assay was performed in 3 independent determinations with multiple preparations of proteins.

### Statistics

Statistical analyses were conducted using GraphPad Prism v7.0. The mean ± standard error of the mean (S.E.M.) is shown in figures, and *P* values were calculated using independent sample t-test, one-way ANOVA or two-way ANOVA, where indicated. A p-value of less than 0.05 was considered statistically significant and is denoted by *. *p* < 0.01 is denoted by **, *p* < 0.001 is denoted by ***, and *p* < 0.0001 is denoted by ****.

### Reporting summary

Further information on research design is available in the Nature Research Reporting Summary linked to this article.

## Supplementary information

Supplementary Information

Description of Additional Supplementary Files

Supplementary Data 1

Reporting Summary

## Data Availability

Raw mass spectrometry data for IP-MS analysis of SopD has been uploaded to the MassIVE repository, accession # MSV000086523 [10.25345/C53497]. All other data that support the findings of this study are available from the corresponding authors upon request. Source data are provided with this paper.

## Source data

Source Data
