# Supplementary material for: Salmonella effector SopD promotes plasma membrane scission by inhibiting Rab10
Source: Nat Commun. 2021 Aug 4;12:4707. doi: 10.1038/s41467-021-24983-z (PMC8339009; doi:10.1038/s41467-021-24983-z)
Supplement: Supplementary file 1 — Supplementary Information [file 41467_2021_24983_MOESM1_ESM.pdf]

Supplementary Information

Salmonella effector SopD promotes plasma membrane scission by inhibiting Rab10

Kirsten C. Boddy[1,2], Hongxian Zhu[1,3], Vanessa M. D'Costa[1,4,5], Caishuang Xu[6], Ksenia Beyrakhova[6], Miroslaw Cygler[6], Sergio Grinstein[1,2,7], Etienne Coyaud[8], Estelle M. N. Laurent[8], Jonathan St-Germain[8], Brian Raught[8,9, Ψ], John H. Brumell[1,2, 3, 10, Ψ, #]

[1]Cell Biology Program, Hospital for Sick Children, Toronto, ON, M5G 1X8, Canada
[2]Institute of Medical Science, University of Toronto, Toronto, ON, M5S 1A8, Canada
[3]Department of Molecular Genetics, University of Toronto, Toronto, ON M5S 1A8, Canada
[4]Department of Biochemistry, Microbiology and Immunology, University of Ottawa, ON, K1H 8M5, Canada
[5]Centre for Infection, Immunity and Inflammation, University of Ottawa, K1H 8M5, Canada
[6]Department of Biochemistry, Microbiology and Immunology, University of Saskatchewan, Saskatoon, SK, S7N 5E5, Canada
[7]Department of Biochemistry, University of Toronto, Toronto, ON M5S1A8, Canada
[8]Princess Margaret Cancer Centre, University Health Network, Toronto, ON, M5S 1A8, Canada
[9]Department of Medical Biophysics, University of Toronto, Toronto, ON M5S 1A8, Canada
[10]SickKids IBD Centre, Hospital for Sick Children, Toronto, ON, M5G 0A4, Canada

[#]Corresponding author:
John H. Brumell, Cell Biology Program, Hospital for Sick Children, 686 Bay Street PGCRL, Toronto, ON, M5G 0A4, Canada Tel: 416-813-7654 ext. 303555. E-mail: john.brumell@sickkids.ca

[Ψ]These authors contributed equally to the manuscript

Supplementary Figures

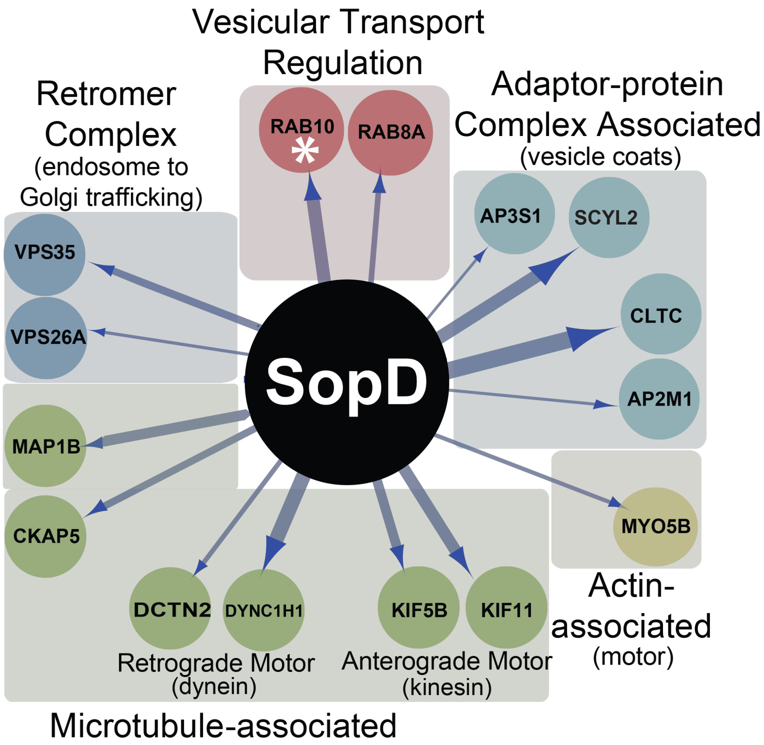

Supplementary Figure 1. SopD endocytic trafficking interactome. Affinity purification of SopD-Flag was followed by mass spectrometry to identify host cell interactors of SopD. Selected interactors and their annotated functions are shown. The thickness of each line represents the strength of the interaction. An asterisk denotes the interactor of interest for this study. Data represents four technical replicates from two biological repeats of the experiment.

```
CLUSTAL 2.1 multiple sequence alignment
SopD
SopD2


MPVTLSFGNHQNYTLNESRLAHLLSADKEKAIHMGGWDKVQDHFRAEKKDHALEVLHSII
MPVTLSFGNRHNYEINHSRLARLMSSDKEEALYMGVWDRFKDCFRTHKKREVLEVLYTLI
*********::** :*.****:*:*:***:*::** **:.:* **:.** ..****:::*

HGQGRGEPGEMEVNVEDINKIYAFKRLQHLACPAHQDLFTIKMDASQTQFLLMVGDTVIS
HGCERENQAELNVDTVGMEKIYAFAQLKQYANPSQQDRFVMRFDMSQTQVSFEIDGKVID
**  * : .*::*:. .::***** :*:: * *::** *.:::* ****. : :...**.

QSNIKDILNISDDTVIESMSREERQLFLQICEVIGSKMTWHPELLQESISTLRKEVTGNA
KCNLHRILNVSENCIFKVMEEDEEELFFKVCIKYGEKIACYPELLENFAFKLRQEVNEDD
:.*:: ***:*:: ::: *..:*.:**:::*   *.*:: :****::   .**:**. :

QIKTAVYEMMRPAEAPDHPLVEWQDLLTADEKSMLACINAGNFEPTTQFCKIGYQEVQGE
EIKDEVYKLMRSGEDRKMACVEWNGTLTEDEMDKLRCLQMGSFEISTQFCKIGYWELEGE
:**  **::**..*  . . ***:. ** ** . * *:: *.** :******** *::**

VAFSMMHPCISYLLHSYSPFAEFKPTNSG---FLKKLNQDYNDYHAKKMFIDVILEKIYL
VLFDMFHPTLIYLLHGYMPSLSCDFTEANTMLFSDVLNKDYDDYQNNKREIDAILRRIYR
* *.*:** : ****.* *  . . *::.   * . **:**:**: :*  **.**.:**

THERSLHIGKDGCSRNILLT
SHNNTLFISEGSCCRNMLL-
:*:.:*.*.:..*.**:**
```

Supplementary Figure 2. Amino Acid sequence alignment of SopD and SopD2.

CLUSTAL 2.1 was used to generate a sequence alignment of SopD (blue) and SopD2 (black). The conserved catalytic arginine residues are indicated (red).

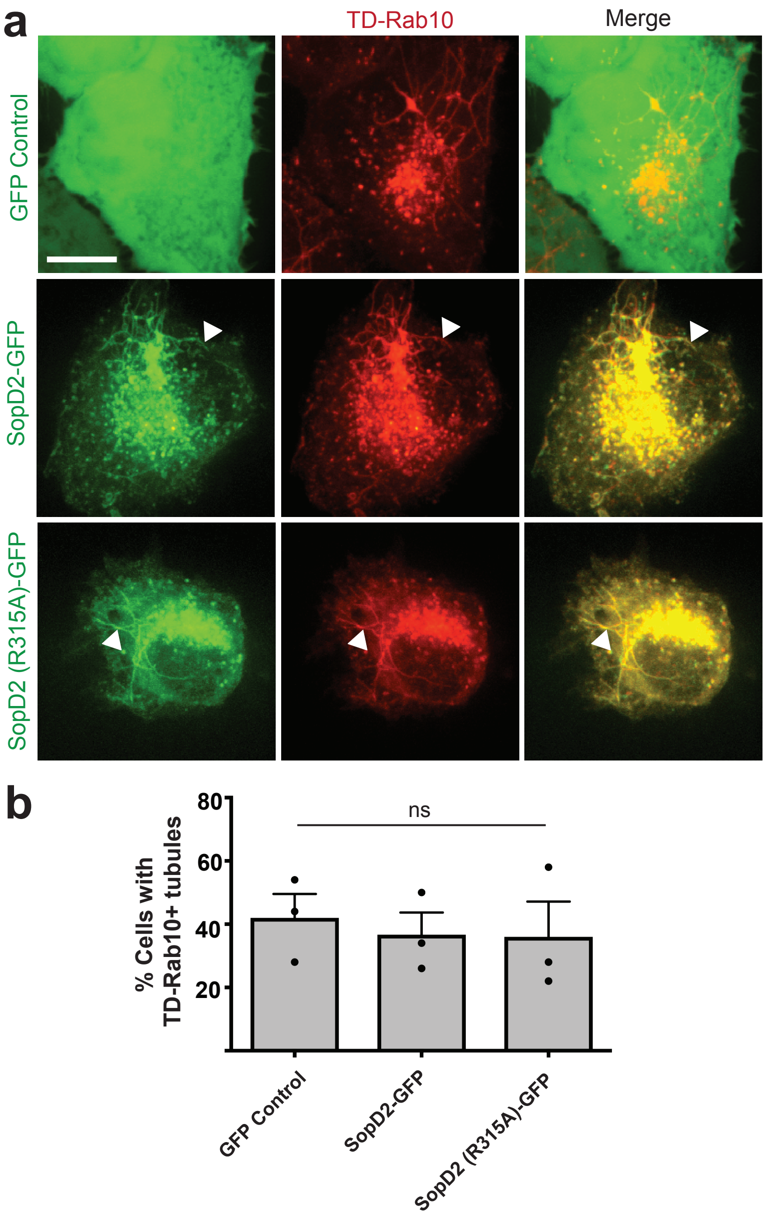

Supplementary Figure 3. SopD2 is not sufficient to disrupt Rab10⁺ tubules in transfected cells. (a) Representative images of Henle 407 cells co-transfected with TD-Rab10 and GFP control, SopD2, or SopD2(R315A)-GFP tagged constructs. Scale Bar, 15 μm. (b) Quantification of the number of co-transfected cells with TD-Rab10$^+$ tubules from (a). In three independent experiments at least 50 cells were scored for the presence or absence of TD-Rab10$^+$ tubules. Data are means ± S.E.M.. Statistical analysis was

performed with a one-way ANOVA. GFP: green fluorescent protein; TD: TdTomato.

Source data are included in source data file.

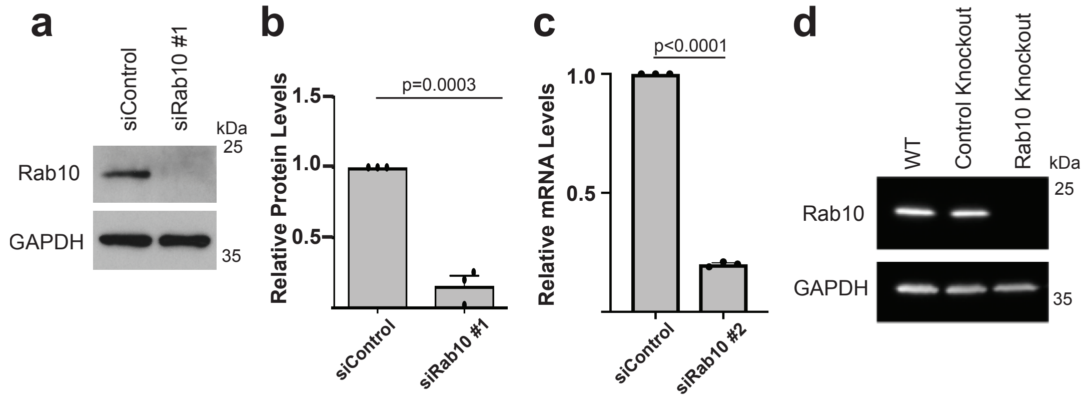

Supplementary Figure 4. Assessment of siRNA mediated knockdown of Rab10 (a-c). Two Rab10 siRNA's were assessed #1 and #2, as described in the methods. Cells were treated with the indicated siRNA for 48 h. (a) Cell lysates were immunoblotted with antibody to endogenous Rab10. GAPDH antibody was used to assess equal protein loading in samples. Data is representative of three independent experiments. (b) Densitometry of three independent experiments was performed using ImageJ. Levels of Rab10 are normalized to GAPDH. Data are means ± S.E.M.. *P* value determined using a two-tailed unpaired Student's *t* test. (c) qRT-PCR was used to assess knockdown of Rab10. Data are means ± S.E.M.. *P* value determined using a two-tailed unpaired Student's *t* test. Source data are included in source data file. **Assessment of CRISPR mediated knockout of Rab10 (d).** Cell lysates from WT, Control knockout and Rab10 knockout Henle 407 cells were immunoblotted with antibody to endogenous Rab10. GAPDH antibody was used to assess equal protein loading in samples. Data is representative of three independent experiments. Source data are included in source data file.

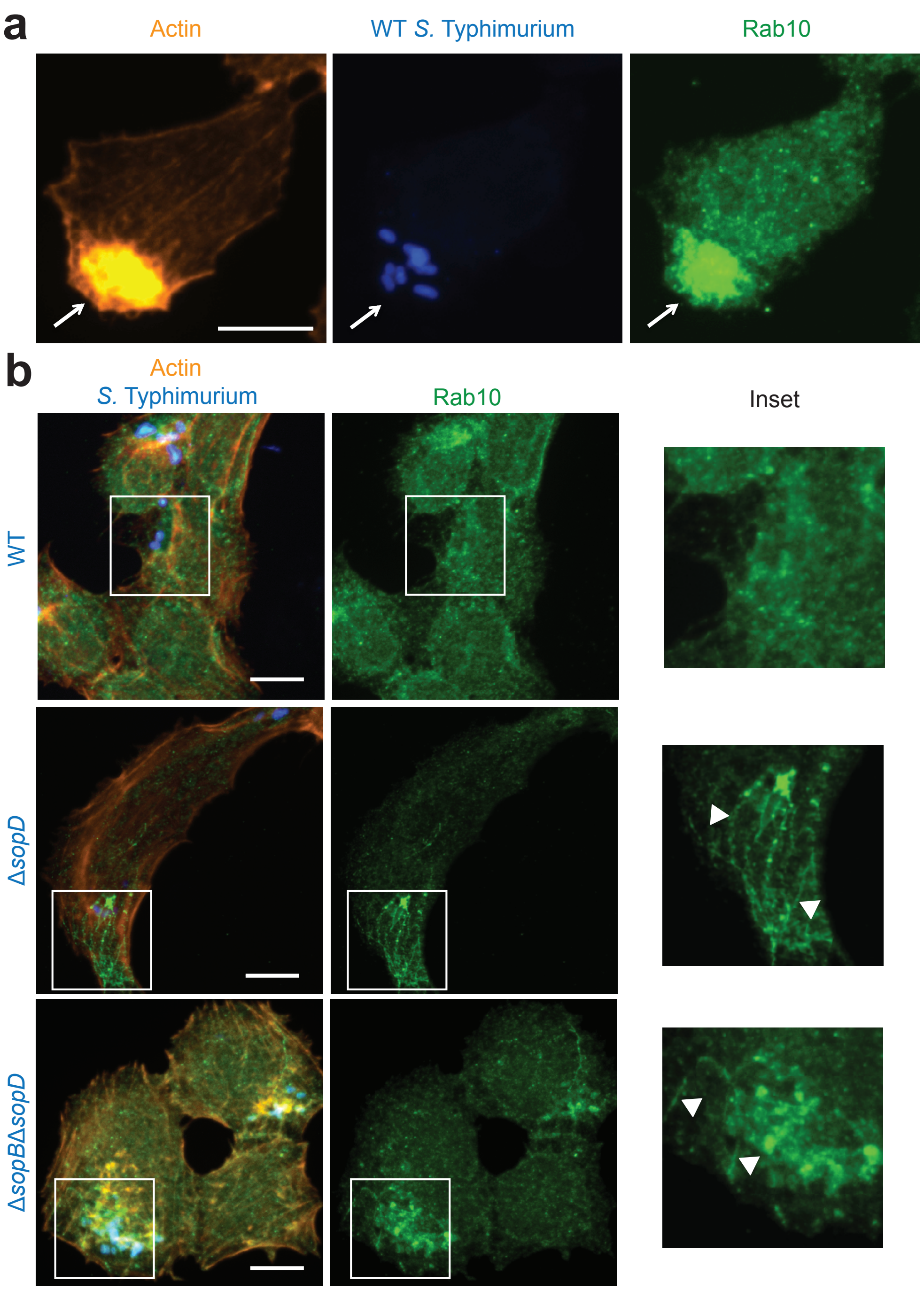

Supplementary Figure 5. Endogenous Rab10 localizes to the *S.* Typhimurium invasion site. (a) Henle 407 cells infected with WT *S.* Typhimurium for 10 min, fixed and stained for actin, *S.* Typhimurium and an antibody targeting Rab10. Arrow indicates invasion site. Data is representative of three independent experiments. Scale Bar, 12 μm.

(b) Representative images of Henle 407 cells infected with the indicated *S.* Typhimurium strain for 30 min. Cells were fixed and stained for actin, *S*. Typhimurium and an antibody targeting Rab10. Arrowheads indicate $Rab10^{+}$ tubules. Scale Bar, 12 μm. Data is representative of three independent experiments. WT: wild type.

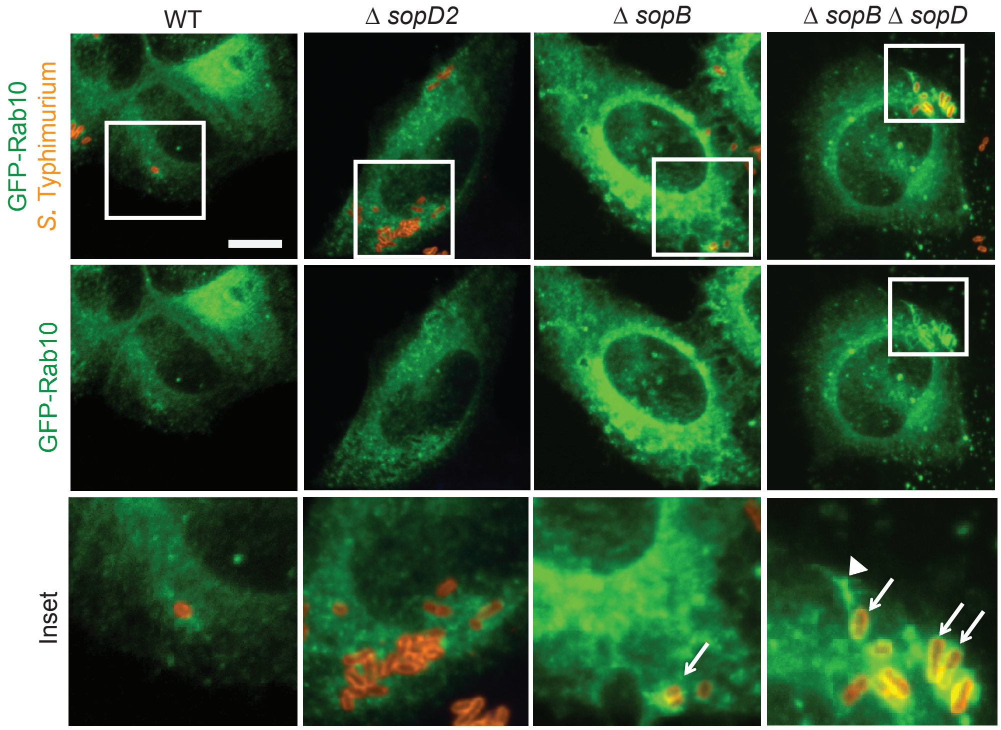

Supplementary Figure 6. SopB and SopD, but not SopD2, disrupts Rab10 trafficking at the *S.* Typhimurium invasion sites. Henle 407 cells were transfected with GFP-Rab10 and infected with strains of *S.* Typhimurium for 30 min. *Salmonella* antibody staining before and after cell permeabilization was used to distinguish bacteria populations undergoing internalization, as described in the Methods section. Shown are representative images of Henle 407 cells transfected with GFP-Rab10 and infected with the indicated strain of *S.* Typhimurium for 30 min. Data is representative of three independent experiments. Scale Bar, 11 µm. GFP: green fluorescent protein; WT: wild type.

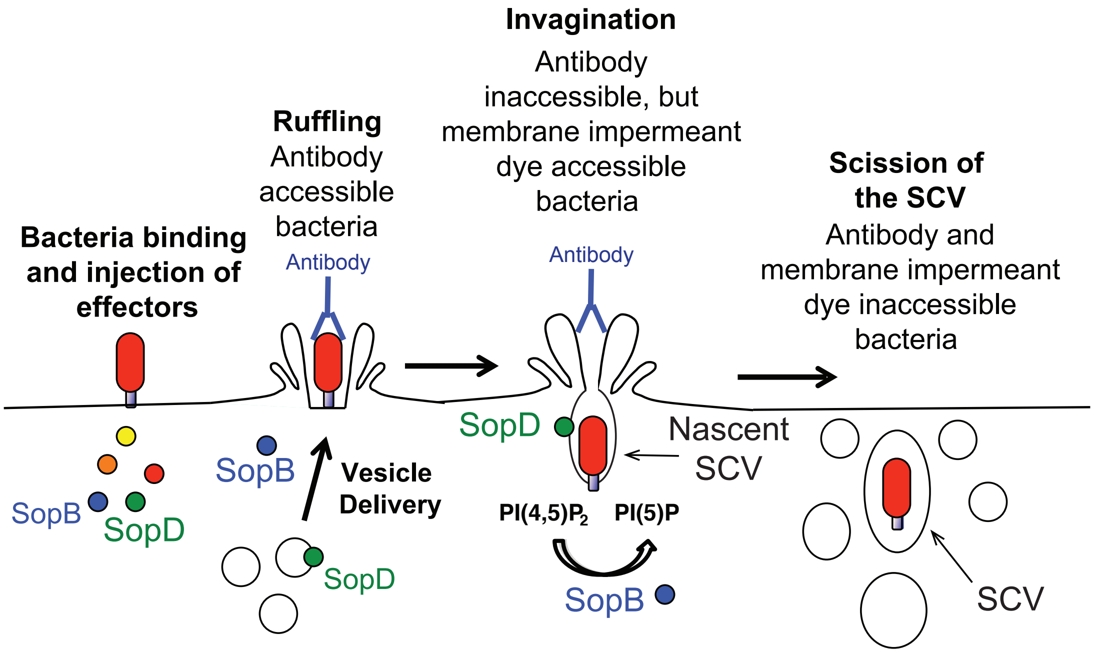

Supplementary Figure 7. Model depicting stages of the *S.* Typhimurium internalization process. In order to distinguish different stages of the invasion process, the accessibility of bacteria to extracellular antibodies and membrane impermeant dyes have been used. During early stages of invasion, bacteria are accessible to extracellular antibody and have not entered a sealed compartment. Subsequently, the bacteria enter a stage of internalization in which they are inaccessible to extracellular antibody but are in compartments that have not fully sealed from the plasma membrane. These bacteria are in compartments that are accessible to membrane impermeant dyes and are referred to as nascent SCV's. Bacteria that are in compartments that have undergone scission from the plasma membrane are in a sealed SCV that is inaccessible to extracellular antibodies and dyes. SCV: *Salmonella*-containing vacuole.

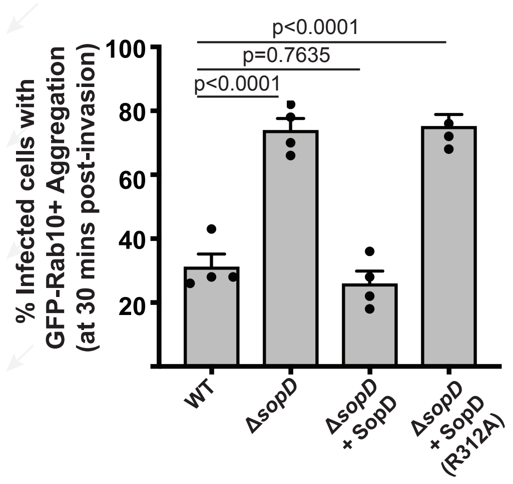

Supplementary Figure 8. The GAP activity of SopD inhibits Rab10 localization at invasion sites. Quantification of the number of Henle 407 cells transfected with GFP-Rab10 and infected with WT and the indicated mutant strains of *S.* Typhimurium for 30 min that had GFP-Rab10$^+$ compartments aggregated at the *S.* Typhimurium invasion site. Data are means ± S.E.M of four independent experiments in which at least 50 invasion sites were scored for the presence or absence of GFP-Rab10$^+$ compartment aggregation. Statistical analysis was performed with a one-way ANOVA. GFP: green fluorescent protein; WT: wild type. Source data are included in source data file.

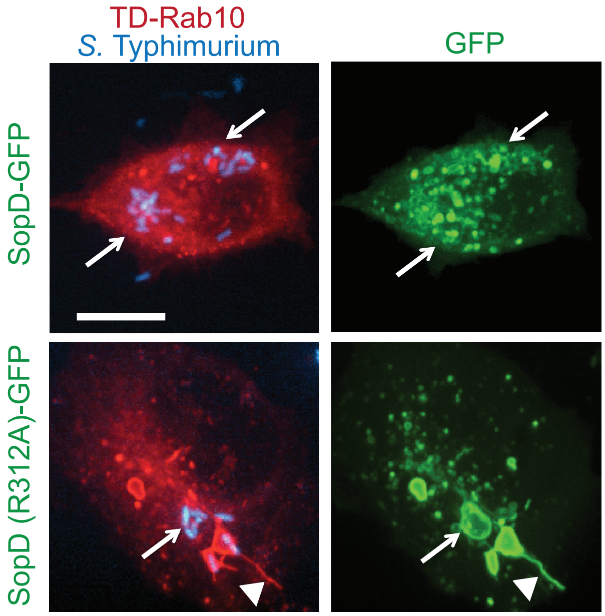

Supplementary Figure 9. SopD GAP mutant co-localizes with TD-Rab10 at the *S.* Typhimurium invasion site. Henle 407 cells were co-transfected with TD-Rab10 and SopD-GFP or SopD(R312A)-GFP tagged constructs, then infected with Δ*sopD* mutant *S.* Typhimurium. Images are representative of live cells ~ 30 min post infection. Arrow indicates GFP^+ bacteria and arrowhead denotes tubules. Data is representative of three independent experiments. Scale Bar, 10 μm. GFP: green fluorescent protein; TD: TdTomato.

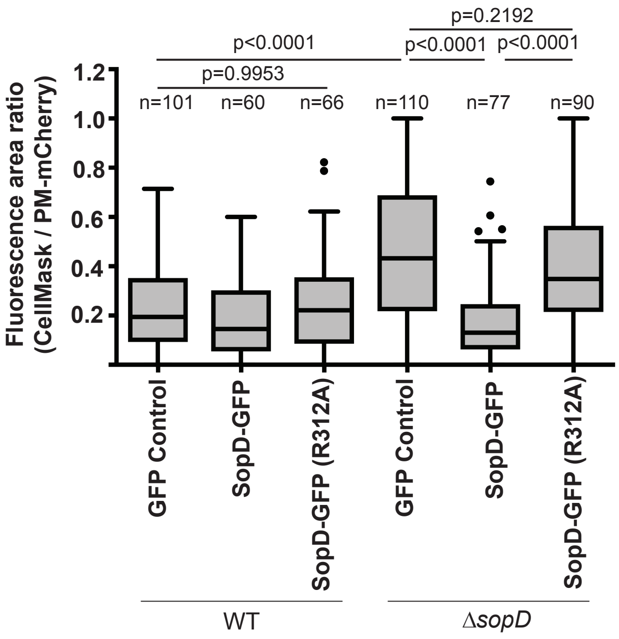

Supplementary Figure 10. SopD can act *in trans* to promote membrane scission during *S.* Typhimurium invasion through its GAP activity. Henle 407 cells were transfected with the indicated GFP construct and PM-mCherry 16-18 h prior to infection with the indicated bacterial strain. Quantification of the extent of membrane scission represented as the fluorescence area ratio. The fluorescence area labelled with CellMask compared to that labelled with PM-mCherry was used as an index of sealing at the invasion site. Data are visualized with Tukey-style boxplots, where the boxes represent the 25th, 50th/median and 75th percentiles. The whiskers denote 1.5× the IQR (interquartile range) from the median. Points denote outliers beyond 1.5× IQR. Statistical analysis was performed with one-way ANOVA. *P*-values and the number of independent cells used for measurements (n) are indicated in the figure. GFP: green fluorescent protein.. Source data are included in source data file.

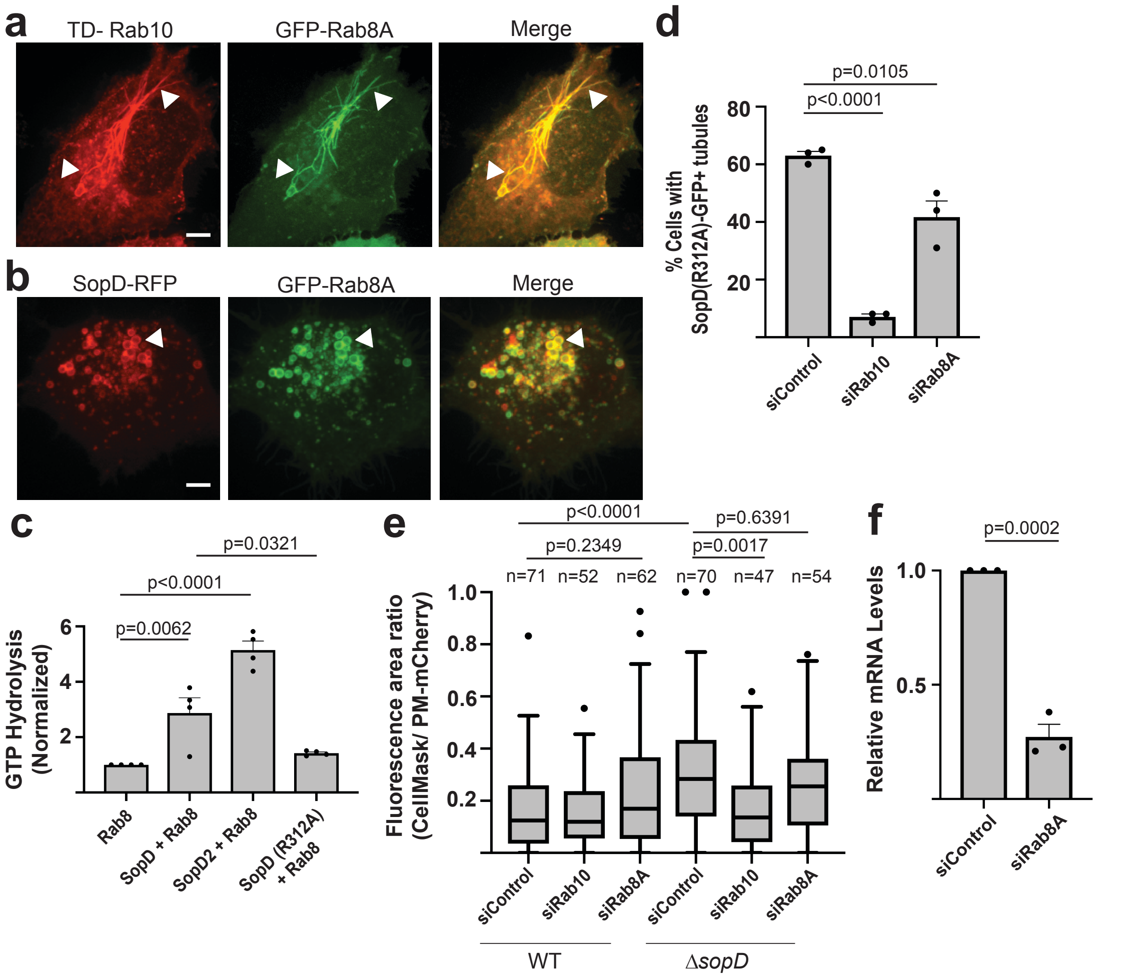

Supplementary Figure 11. Rab8A is not a relevant target of SopD during *S.* Typhimurium invasion. (a) Representative images of Henle 407 cells co-transfected with TD-Rab10 and GFP-Rab8A, arrowheads indicate colocalization. Scale Bar, 5 µm. Data is representative of three independent experiments. (b) Representative images of Henle 407 cells co-transfected with SopD-RFP and GFP-Rab8A, arrowheads indicate colocalization. Scale Bar, 5 µm. Data is representative of three independent experiments. (c) Purified Rab8 (8 µM) was incubated alone and with SopD, SopD2 or the SopD catalytic mutant (R312A) (8 µM) and the GTP hydrolysis is represented relative to Rab8 alone. GTP hydrolysis was measured by a malachite green assay as indicated in the methods. Data represents relative GTP hydrolysis and are the mean ± S.E.M. of 6

technical replicates from 3 independent determinations with multiple protein preparations. Statistical analysis was performed with a one-way ANOVA. (d) Henle 407 cells were transfected with the indicated siRNA and then SopD(R312A)-GFP 24 h later. The number of transfected cells with SopD(R312A)$^{+}$ tubules were quantified. In three independent experiments at least 100 cells were scored. Data are means ± S.E.M.. Statistical analysis was performed with one-way ANOVA. (e) Henle 407 cells were transfected with the indicated siRNA and then PM-mCherry 24 h later. The extent of membrane scission in cells infected with WT and *ΔsopD* mutant *Salmonella* is represented as the fluorescence area ratio. The fluorescence area labelled with CellMask compared to that labelled with PM-mCherry was used as an index of sealing at the invasion site. Data are visualized with Tukey-style boxplots, where the boxes represent the 25th, 50th/median and 75th percentiles. The whiskers denote 1.5× the IQR (interquartile range) from the median. Points denote outliers beyond 1.5× IQR. Statistical analysis was performed with one-way ANOVA. *P*-values and the number of independent cells used for measurements (n) are indicated in the figure. (f) qRT-PCR was used to assess knockdown of Rab8A. Data are means ± S.E.M. of three independent experiments. *P*-value determined using a two-tailed unpaired Student's *t* test. WT: wild type; RFP: red fluorescent protein; GFP: green fluorescent protein; TD: TdTomato. Source data are included in source data file.

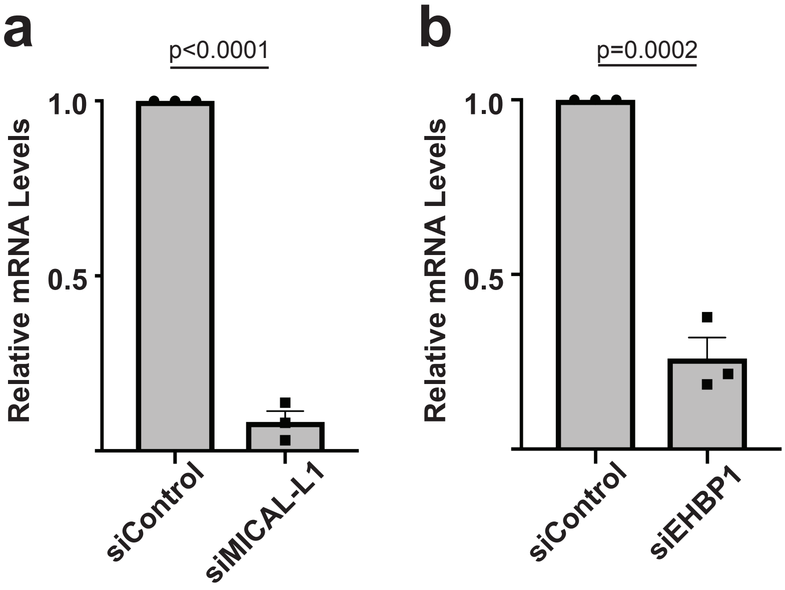

Supplementary Figure 12. Assessment of siRNA mediated knockdown of Rab10 effectors. (a) qRT-PCR was used to assess knockdown of MICAL-L1. Data are means ± S.E.M. of three independent experiments. *P* value determined using a two-tailed unpaired Student's *t* test. (b) qRT-PCR was used to assess knockdown of EHBP1. Data are means ± S.E.M. of three independent experiments. *P*-value determined using a two-tailed unpaired Student's *t* test. Source data are included in source data file.

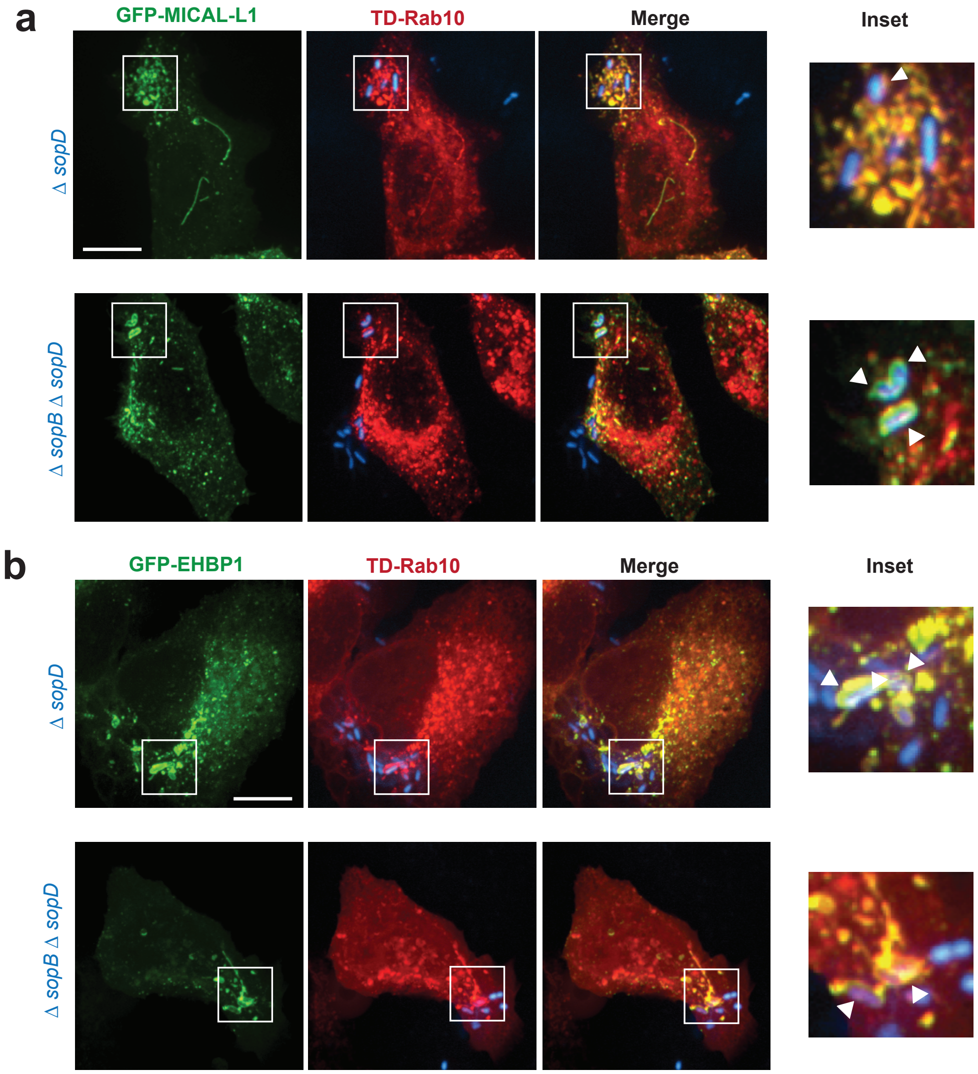

Supplementary Figure 13. Rab10 effectors co-localize with Rab10 during invasion with Δ*sopD* mutant *Salmonella*. (a) Representative images of Henle 407 cells co-transfected with TD-Rab10 and GFP-MICAL-L1 then infected with the indicated mutant *S.* Typhimurium strain for 20 min. Arrowheads denote colocalization. Scale Bar, 10 μm. Data is representative of three independent experiments. (b) Representative images of Henle 407 cells co-transfected with TD-Rab10 and GFP-EHBP1 then infected with the indicated mutant *S.* Typhimurium strain for 20 min. Arrowheads denote colocalization.

Scale Bar, 10 μm. Data is representative of three independent experiments. GFP: green fluorescent protein; TD: TdTomato.

Supplementary Tables

Supplementary Table 1. Primers used in this study.

Primer	Sequence
KBP1	TATAGGCGCGCCATGCCAGTCACTTTAAGCTTCG
KBP2	TTAAGCGGCCGCTGTCAGTAATATATTACGACTGCACC
KBP3	CTAATATATTACTGACGGATCCACCGGTCGC
KBP4	CACTGCACCCATCTTTACCAATGTGCAAAG
KBP5	CTAATATATTACTGACACTCGAGTATCCGTATGATGTGC
KBP6	CACTGCACCCATCTTTACCAATGTGCAAAG
KBP7	TGCAGGATCCGCCACCATGGGCTGCATTAAAAGCAAACGCAAAGATATGGTGAGCAAGG<br>GCGAGGAGGATAACATG
KBP8	TGCAGCGGCCGCTTACTTGTACAGCTCGTCCATGCCGCCGGT
KBP9	GATGGGTGCAGTGCTAATATATTACTGAC
KBP10	GTCAGTAATATATTAGCACTGCACCCATC
KBP11	TACTTCCAATCCAA TGCCAAGACCTACGATTACCTGTTCAAGCTG
KBP12	TTATCCACTTCCAATGTTACCCCTGGGGGCTGTTGCCTTCC
KBP13	GGATCCTCTAGATTTAAGAAGGAGATATACATATGAGCGAGCTGATTAAGGAGAAC
KBP14	CTTGCATGCCTGCAGGAGATTTAATTAAGCTTGTGCCCCAGTTTG
KBP15	AGGAAGAAGGTACCGCGGGCCCGGGATCCATGAGCGAGCTGATTAAGGAGAAC
KBP16	GGTATGGCTGATTATGATCAGTTATCTAGATTAATTAAGCTTGTGCCCCAGTTTG
