# Supplementary material for: Salmonella effector SopD promotes plasma membrane scission by inhibiting Rab10
Source: Nat Commun. 2021 Aug 4;12:4707. doi: 10.1038/s41467-021-24983-z (PMC8339009; doi:10.1038/s41467-021-24983-z)
Supplement: Supplementary file 2 — Description of Additional Supplementary Files [file 41467_2021_24983_MOESM2_ESM.pdf]

Description of Additional Supplementary Files

File Name: Supplementary Data 1

Description: IP-MS report of SopD interactors. Individual preys and their presence or absence in SopD and control runs are shown. Data represents four technical replicates from two biological replicates of the experiment. Total peptide counts are shown for each replicate. Statistical significance is represented as a SAINT score.
