# Supplementary material for: Salmonella effector SopD promotes plasma membrane scission by inhibiting Rab10
Source: Nat Commun. 2021 Aug 4;12:4707. doi: 10.1038/s41467-021-24983-z (PMC8339009; doi:10.1038/s41467-021-24983-z)
Supplement: Supplementary file 4 — Reporting Summary [file 41467_2021_24983_MOESM4_ESM.pdf]

nature research

Corresponding author(s): John Brumell

Last updated by author(s): July 13, 2021

Reporting Summary

Nature Research wishes to improve the reproducibility of the work that we publish. This form provides structure for consistency and transparency in reporting. For further information on Nature Research policies, see our Editorial Policies and the Editorial Policy Checklist.

Statistics

For all statistical analyses, confirm that the following items are present in the figure legend, table legend, main text, or Methods section.

n/a	Confirmed	
☐	☒	The exact sample size (*n*) for each experimental group/condition, given as a discrete number and unit of measurement
☐	☒	A statement on whether measurements were taken from distinct samples or whether the same sample was measured repeatedly
☐	☒	The statistical test(s) used AND whether they are one- or two-sided<br>*Only common tests should be described solely by name; describe more complex techniques in the Methods section.*
☐	☒	A description of all covariates tested
☐	☒	A description of any assumptions or corrections, such as tests of normality and adjustment for multiple comparisons
☐	☒	A full description of the statistical parameters including central tendency (e.g. means) or other basic estimates (e.g. regression coefficient) AND variation (e.g. standard deviation) or associated estimates of uncertainty (e.g. confidence intervals)
☐	☒	For null hypothesis testing, the test statistic (e.g. *F*, *t*, *r*) with confidence intervals, effect sizes, degrees of freedom and *P* value noted<br>*Give P values as exact values whenever suitable.*
☒	☐	For Bayesian analysis, information on the choice of priors and Markov chain Monte Carlo settings
☒	☐	For hierarchical and complex designs, identification of the appropriate level for tests and full reporting of outcomes
☒	☐	Estimates of effect sizes (e.g. Cohen's *d*, Pearson's *r*), indicating how they were calculated

Our web collection on statistics for biologists contains articles on many of the points above.

Software and code

Policy information about availability of computer code

Data collection	Volocity 6.3 acquisition software (Improvision) was used for confocal microscopy.
Data analysis	Volocity 6.3 software (Improvision) was used for analysis of Salmonella invasion sites and bacteria-containing vacuoles.. Statistical analyses were conducted using GraphPad Prism v7.0

For manuscripts utilizing custom algorithms or software that are central to the research but not yet described in published literature, software must be made available to editors and reviewers. We strongly encourage code deposition in a community repository (e.g. GitHub). See the Nature Research guidelines for submitting code & software for further information.

Data

Policy information about availability of data

All manuscripts must include a data availability statement. This statement should provide the following information, where applicable:

- Accession codes, unique identifiers, or web links for publicly available datasets
- A list of figures that have associated raw data
- A description of any restrictions on data availability

The authors declare that all the relevant data supporting the findings of the study are available in this article and its Supplementary Information files. Raw mass spectrometry data for IP-MS analysis of SopD has been uploaded to the MassIVE repository, accession # MSV000086523 [doi:10.25345/C53497].

Field-specific reporting

Please select the one below that is the best fit for your research. If you are not sure, read the appropriate sections before making your selection.

☒ Life sciences ☐ Behavioural & social sciences ☐ Ecological, evolutionary & environmental sciences

For a reference copy of the document with all sections, see nature.com/documents/nr-reporting-summary-flat.pdf

Life sciences study design

All studies must disclose on these points even when the disclosure is negative.

Sample size	For cell biological studies, we followed generally accepted standards in the field for sample size determination.
Data exclusions	Not applicable
Replication	All cell biological experiments were performed in three independent biological replicates. For mass spectrometry studies, at least two biological replicates were performed for each experimental condition and two technical replicates were analyzed by mass spectrometry for each biological replicate.
Randomization	Not applicable for cell biological studies.
Blinding	Where possible, investigators were blinded during data analysis of microscopy studies where positive- and negative- scoring was performed.

Reporting for specific materials, systems and methods

We require information from authors about some types of materials, experimental systems and methods used in many studies. Here, indicate whether each material, system or method listed is relevant to your study. If you are not sure if a list item applies to your research, read the appropriate section before selecting a response.

Materials & experimental systems

n/a	Involved in the study
☐	☒ Antibodies
☐	☒ Eukaryotic cell lines
☒	☐ Palaeontology and archaeology
☒	☐ Animals and other organisms
☒	☐ Human research participants
☒	☐ Clinical data
☒	☐ Dual use research of concern

Methods

n/a	Involved in the study
☒	☐ ChIP-seq
☒	☐ Flow cytometry
☒	☐ MRI-based neuroimaging

Antibodies

Antibodies used	Rabbit polyclonal anti-S. Typhimurium LPS (Difco, polyclonal, Cat #229481 , Lots #4017189 and 6085528), mouse monoclonal anti-RAB10 (Sigma, Cat # SAB5300028, Lot # PM1009301), mouse monoclonal anti-GFP (Molecular Probes, clone 3E6, Cat # A11120, Lots # 939305, 1563696 and 1711553), chicken polyclonal anti-GFP (Rockland polyclonal, Cat #600-901-215 , Lot #32390), rabbit polyclonal anti-GFP (Molecular Probes, polyclonal, Cat #A-11122, Lot #1296649), rat monoclonal anti-RFP (Chromotek, clone 5F8, Cat #5f8 , Lots #110411 and 60706002AB), mouse monoclonal anti-GAPDH (Millipore, Cat #MAB374, Lot #3126954).
Validation	Rabbit polyclonal anti-S. Typhimurium LPS (validated by manufacturer and in literature for immunofluorescence), mouse monoclonal anti-RAB10 (validated by manufacturer for immuno blotting and immunofluorescence), mouse monoclonal anti-GFP (validated by manufacturer and in literature for immunofluorescence), chicken polyclonal anti-GFP (validated by manufacturer for immunofluorescence), rabbit polyclonal anti-GFP (validated by manufacturer and in literature for immunofluorescence and immunoblotting), rat monoclonal anti-RFP (validated by manufacturer and in literature for immunofluorescence and immunoblotting), mouse monoclonal anti-GAPDH (validated by manufacturer and in literature for and immunoblotting).

Eukaryotic cell lines

Policy information about cell lines

Cell line source(s)	Henle 407 cells were obtained from the American Type Culture Collection (ATCC), HEK 293 Flp-In T-REx were from Invitrogen.
Authentication	As all cell lines were obtained directly from suppliers, authentication was not performed.

Mycoplasma contamination	All parent cell lines were tested for Mycoplasma upon receipt from manufacturer, and results were negative.
Commonly misidentified lines<br>(See ICLAC register)	Not applicable
